# The TRAPPC8/TRS85 subunit of the Arabidopsis TRAPPIII tethering complex regulates endoplasmic reticulum function and autophagy

**DOI:** 10.1093/plphys/kiaf042

**Published:** 2025-03-14

**Authors:** Marta Hoffman-Sommer, Natalia Piłka, Anna Anielska-Mazur, Julita Nowakowska, Małgorzata Kozieradzka-Kiszkurno, Cezary Pączkowski, Małgorzata Jemioła-Rzemińska, Kamil Steczkiewicz, Yasin Dagdas, Ewa Swiezewska

**Affiliations:** Polish Academy of Sciences, Institute of Biochemistry and Biophysics, ul. Pawinskiego 5a, Warsaw 02-106, Poland; Polish Academy of Sciences, Institute of Biochemistry and Biophysics, ul. Pawinskiego 5a, Warsaw 02-106, Poland; Polish Academy of Sciences, Institute of Biochemistry and Biophysics, ul. Pawinskiego 5a, Warsaw 02-106, Poland; Faculty of Biology, University of Warsaw, ul. Miecznikowa 1, Warsaw 02-096, Poland; Faculty of Biology, University of Gdańsk, ul. Wita Stwosza 59, Gdańsk 80-308, Poland; Faculty of Biology, University of Warsaw, ul. Miecznikowa 1, Warsaw 02-096, Poland; Department of Plant Physiology and Biochemistry, Faculty of Biochemistry, Biophysics and Biotechnology, Jagiellonian University, Gronostajowa 7, Kraków 30-387, Poland; Polish Academy of Sciences, Institute of Biochemistry and Biophysics, ul. Pawinskiego 5a, Warsaw 02-106, Poland; Austrian Academy of Sciences, Vienna BioCenter, Gregor Mendel Institute, Dr. Bohr-Gasse 3, Vienna 1030, Austria; Polish Academy of Sciences, Institute of Biochemistry and Biophysics, ul. Pawinskiego 5a, Warsaw 02-106, Poland

## Abstract

Transport protein particle (TRAPP) tethering complexes are known for their function as Rab GTPase exchange factors. Two versions of the complex are considered functionally separate: TRAPPII, an activator of the Rab11 family (RabA in plants) GTPases that function in post-Golgi sorting, and TRAPPIII, activating Rab1 family (RabD in plants) members that regulate endoplasmic reticulum (ER)-to-Golgi trafficking and autophagy. In Arabidopsis (*Arabidopsis thaliana*), the TRAPPIII complex has been identified and its subunit composition established, but little is known about its functions. Here, we found that binary subunit interactions of the plant TRAPPIII complex are analogous to those of metazoan TRAPPIII, with the 2 large subunits TRAPPC8 and TRAPPC11 linking the TRAPP core and the small C12 to C13 dimer. To gain insight into the functions of TRAPPIII in plants, we characterized 2 *A. thaliana trappc8* mutants. These mutants display abnormalities in plant morphology, particularly in flower and seed development. They also exhibit autophagic defects, a constitutive ER stress response, and elevated levels of the ER lipid dolichol (Dol), which is an indispensable cofactor in protein glycosylation. These results indicate that plant TRAPPC8 is involved in multiple cellular trafficking events and suggest a link between ER stress responses and Dol levels.

## Teaser

Loss of a tethering complex subunit causes defective secretion and autophagosome formation, higher dolichol levels, and aberrant plant development, indicating its role in endoplasmic reticulum function.

## Introduction

Intracellular membrane traffic is critically important for the correct functioning of all eukaryotic cells because it maintains organelle identity. The existence of separate compartments in a cell is dependent on the tight regulation of transport events, which must ensure the delivery of various compounds from the sites of their synthesis or entry into the cell to precisely defined destination sites (the sites of their activity and/or deposition). Rab GTPases play a crucial role in these processes: they contribute to membrane identity and they provide specificity by recruiting appropriate effector proteins (Pfeffer 2013; Minamino and Ueda 2019; Elliott et al. 2020; Nielsen 2020). Localization of a specific Rab to the correct membrane and its activation is dependent on a broad set of regulatory proteins, among which are guanine-nucleotide exchange factors (GEFs) (Lamber et al. 2019), which activate their cognate Rabs by catalyzing the exchange of a Rab-bound GDP molecule for a fresh GTP molecule.

The TRAnsport Protein Particle (TRAPP) complexes TRAPPII and -III both function as Rab GEFs for the Ypt1/Rab1 or/and Ypt31/32/Rab11 families. They differ from each other by subunit composition and substrate specificity toward members of the 2 Rab families. In yeast, TRAPPIII activates Ypt1p (Thomas et al. 2018) while TRAPPII activates Ypt31p/32p (Thomas and Fromme 2016); in animal cells, TRAPPII likely activates members of both Rab families, while TRAPPIII is Rab1-specific (Riedel et al. 2018; Harris et al. 2021). The TRAPP complexes are conserved in eukaryotes from yeast to animals and plants, though higher eukaryotes and some fungi have additional subunits in TRAPPIII, absent in yeast cells (TRAPPC11, −12, −13) (Choi et al. 2011; Scrivens et al. 2011; Bassik et al. 2013; Pinar et al. 2019), and one TRAPPIII subunit (TRAPPC8) has only partial similarity to its yeast counterpart, Trs85p. Further, a plant-specific component has been identified in TRAPPII (TRIPP; Garcia et al. 2020), showing that the plant and animal complexes also differ from each other.

**Figure 1.**
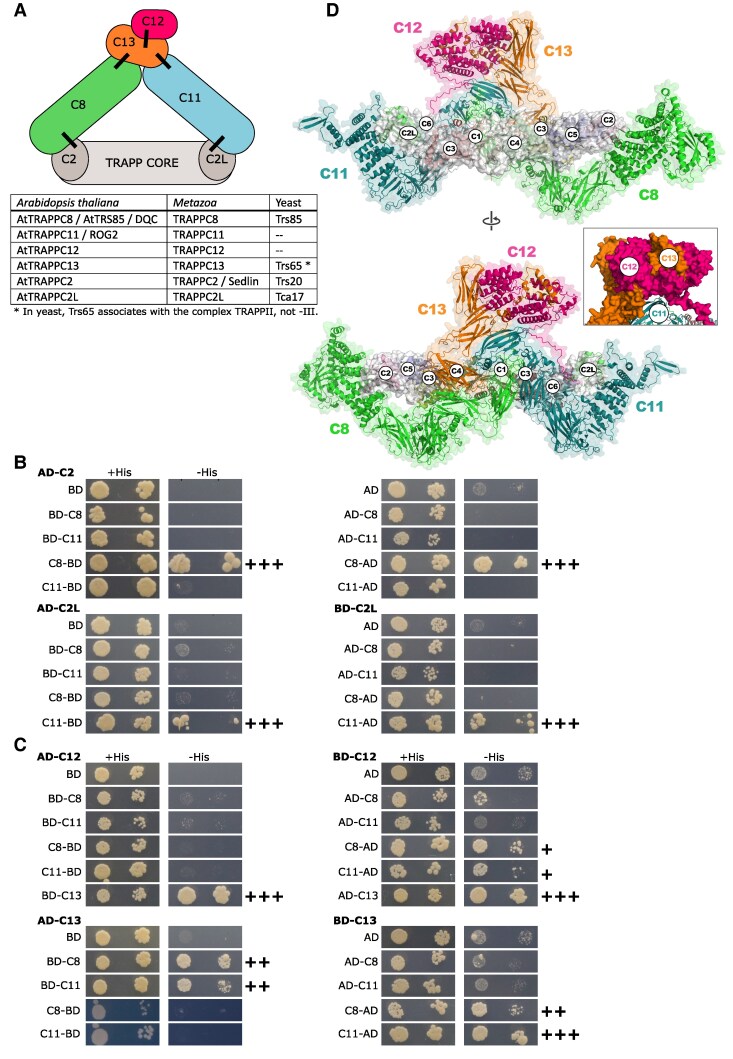
Overall architecture of *A. thaliana* TRAPPIII. **A)** Schematic depiction of relations between TRAPPIII subunits and nomenclature of subunits between kingdoms. Interactions confirmed by the Y2H assay are indicated with black lines. Subunit coloring as in **D)**. **B and C)** Binary interactions between selected TRAPP subunits assayed using the yeast Y2H system. For each panel, one hybrid protein is indicated above the panel and the other to the left. Abbreviations used: AD, Gal4-activation domain; BD, Gal4 DNA-binding domain. The names of TRAPP subunits are shortened to subunit numbers. +His marks control plates, −His are test plates showing reporter activation. Interactions scored as positive (when compared with the relevant control interactions) are indicated by plus signs next to the images: +++ strong growth at highest dilution, ++ medium growth at highest dilution, +small colonies at highest dilution. **B)** Interactions of TRAPPC8 and TRAPPC11 with the adaptor subunits TRAPPC2 and TRAPPC2L. **C)** Interactions of TRAPPC12 and TRAPPC13 with each other and with the large subunits TRAPPC8 and TRAPPC11. **D)** Theoretical structural model of full TRAPPIII from *A. thaliana*. The names of TRAPP subunits are shortened to subunit numbers.

In this work, we concentrated on TRAPPIII. The structures of yeast and metazoan TRAPPIII have been solved (Tan et al. 2013; Galindo et al. 2021; Joiner et al. 2021), so the positioning of the additional metazoan subunits TRAPPC8, −11, −12, −13 in relation to the common TRAPP core is known (see Fig. 1A). In the fruit fly *Drosophila melanogaster*, the large subunits C8 and C11 are in proximity of each other on one end and attach to the TRAPP core on their opposing ends (with C8 connecting to the core through the subunit C2, and C11 through C2L), while the smaller subunits C12 and C13 form a heterodimer that attaches to the C8–C11 joint. Rab1 binding by the TRAPP core is thought to be facilitated by the conserved N-terminal arm of the C8 subunit (Galindo et al. 2021). For yeast and animal TRAPPIII, some functional data is also available. The yeast (*Saccharomyces cerevisiae*) Trs85p protein has homology to animal and plant TRAPPC8 over the N-terminal half of the protein but lacks the C-terminal part that in *D. melanogaster* extends toward C11 and the C12–C13 dimer. It has long been known that yeast TRAPP functions in transport from the endoplasmic reticulum to the Golgi apparatus (ER-to-Golgi) (Sacher et al. 1998, 2001), and that *trs85*Δ cells also have a defect in the Cvt pathway (*cytoplasm-to-vacuole targeting*, a selective, autophagy-related transport pathway that delivers cytoplasmic cargo directly to the vacuole, where it is destined to perform its functions) and impaired (though not completely blocked) autophagy (Meiling-Wesse et al. 2005; Nazarko et al. 2005; Lynch-Day et al. 2010). A function in the secretory pathway at the ER-to-Golgi stage, in Golgi organization, and autophagy has also been later shown for metazoan TRAPPC8 (Scrivens et al. 2011; Lamb et al. 2016; Zhao et al. 2017), C11 (Stanga et al. 2019), and C13 (Ramírez-Peinado et al. 2017). In particular, the C8 subunit has been shown not only to contain the Rab1-binding site (Galindo et al. 2021) but also a membrane-binding site (Harris et al. 2021). Since activation of a specific Rab can take place only on the correct membrane, where its activity is needed, this makes TRAPPC8 a key subunit for the GEF activity of the complex. Additionally, animal C8 and C12 have been implicated in ciliogenesis and the functioning of cilia (Schou et al. 2014; Zhang et al. 2020), while TRAPPC11 has been shown to play a role in protein glycosylation (DeRossi et al. 2016; Matalonga et al. 2017; Larson et al. 2018; Munot et al. 2022; Corona-Rivera et al. 2024).

Plant TRAPPIII is not as well described as the yeast and metazoan complexes. Its subunit composition has been analyzed (Kalde et al. 2019), but no structural data has been collected for the *Arabidopsis* complex to date. Furthermore, open questions remain concerning its putative GEF functions. Plant TRAPPC11 has been found in *trans*-Golgi/early endosome (TGN/EE) vesicles (Drakakaki et al. 2012; Rosquete et al. 2019b), where it colocalized with core TRAPP components, the TRAPPC8 subunit, and the RABD2 GTPases. Data available for the C11 subunit of TRAPPIII suggest a possible role as a membrane tether at the TGN/EE (Rosquete et al. 2019a, b), possibly as a GEF for RABD2. In this scenario, TRAPPIII would be involved in Golgi and post-Golgi trafficking as well as endocytic sorting and recycling of specific components. Song et al. (2020) have isolated and analyzed *Arabidopsis thaliana* insertion mutant lines with mutations in the AtTRAPPC8-encoding gene (AT5G16280). They observed defects in root development and morphology and, therefore, looked at cellular-level changes in root cells, finding mislocalization of the auxin efflux carrier PIN-formed 1 (PIN1) as well as of the TGN/EE proteins RABD2A and VTI12, and abnormalities in the morphology and integrity of compartments of the secretory and endocytic pathways: the *trans*-Golgi network, early endosomes, and vacuoles. A report by Allen et al. (2024) demonstrates a requirement for AtTRAPPC8 in both exo- and endocytosis of cellulose synthase complexes. Another potential function was suggested by Kalde et al. (2019), who showed the binding of TRAPPIII to the vacuole-related RABG3F protein. Together, these reports indicate a wide variety of functions for TRAPPIII components in the cells of higher eukaryotes.

In this study, we focused on the *A. thaliana* TRAPPC8 subunit. In preliminary studies, we isolated plant lines with mutations in all 4 TRAPPIII-specific subunits (C8, −11, −12, and −13), and of these, we chose *trappc8* mutants for analysis due to their most pronounced phenotypes. First, by means of the yeast 2-hybrid (Y2H) system, we positioned TRAPPC8 in the network of intracomplex interactions with other TRAPPIII subunits, and we found a strong analogy to the architecture of the metazoan complex. Next, we performed in-depth phenotypic characterization of *trappc8* plants, establishing a role for this protein in flower and seed development. Finally, we looked at specific cellular processes known to be affected by *trappc8* mutations in other organisms, and we show the engagement of *Arabidopsis* TRAPPC8 in autophagy and ER functioning, including a role in the modulation of dolichol (Dol) levels.

## Results

### Binary interactions in the plant TRAPPIII complex are consistent with an architecture similar to that of metazoan TRAPPIII

The structure of the plant TRAPPIII complex has not been investigated, but the subunit composition has been established (Kalde et al. 2019; Rosquete et al. 2019b) and resembles that of metazoan TRAPPIII, whose structure has already been investigated (Galindo et al. 2021). To see if the complexes can be considered structurally similar, we investigated binary interactions among the TRAPPIII-specific (C8, C11, C12, C13) and adaptor (C2, C2L) subunits from *A. thaliana* using the yeast Y2H system (for subunit nomenclature see table in Fig. 1A).

We concentrated on the interactions of the 2 large subunits, TRAPPC8 and C11. For these proteins, we prepared both N-terminal and C-terminal fusions with the GAL4-activation domain (AD) and GAL4-binding domain (BD) and tested all possible binary interactions with N-terminal AD and BD fusions of the C2, C2L, C12, and C13 subunits. Consistent with the metazoan structure (Galindo et al. 2021), which shows that the N-terminal parts of the C8 and C11 subunits are involved in interactions with the adaptor proteins, the C-terminal fusions displayed clear interactions with the C2 and C2L subunits (Fig. 1B): C8 with C2 and C11 with C2L, while the N-terminal fusions could not interact with the adaptor subunits. When assayed together, the C8 and C11 subunits did not interact with each other in any combination (Supplementary Fig. S1), which is also consistent with intersubunit chemical cross-linking data that was obtained previously for the *Drosophila* TRAPPIII complex (Galindo et al. 2021), supporting the conclusion that their interaction requires the presence of the subunits C12 and/or C13.

The C12 and C13 subunits interacted strongly with each other in all combinations, confirming the presence of a C12–C13 dimer. This confirms that TRAPPC13 is part of the complex (as indicated before by the results of Kalde et al. 2019), unlike the yeast protein Trs65p (homolog of TRAPPC13), which is part of TRAPPII (Choi et al. 2011). Interactions with the large subunits presented a less clear picture: C12 displayed only a weak and 1-way interaction with the C-terminal C8 and C11 fusions, while the C13 subunit had stronger, 2-way interactions with both large subunits (Fig. 1C). This suggests that in *Arabidopsis* C13 might be mediating the connection between the dimer and the C8 and C11 subunits. In the case of the *Drosophila* complex, the C11 subunit—but not C8—could be crosslinked to both C12 and C13 (Galindo et al. 2021).

We then prepared a theoretical model of the full *Arabidopsis* TRAPPIII complex (Fig. 1D) and compared the predicted structure with the identified Y2H interactions. In agreement with the Y2H data, the modeling predicts only a minor interaction interface between C8 and C11 in the plant structure, and interactions of both large subunits with C13 but not C12 (see inset in Fig. 1D). For the subunits C8 and C11, we also compared sets of plant versus animal amino acid sequences to search for differences in domain architecture, but high conservation was found throughout the sequences (Supplementary Fig. S2A). The model of plant TRAPPIII aligned well with the published cryo-EM density map for *Drosophila* (Galindo et al. 2021). We compared the structural models for *A. thaliana* and *D. melanogaster* TRAPPIII and found only minor differences between the structures (e.g. the additional fragment at the N-terminus of *Drosophila* TRAPPC11 that extends outward—however, this additional fragment is present only in a subset of animal sequences) (Supplementary Fig. S2B). These results underscore the high degree of structural conservation of TRAPPIII between plants and animals.

### Loss of Arabidopsis TRAPPC8 leads to smaller plants with excessive branching and defective siliques

To learn more about the in vivo functions of plant TRAPPIII, we decided to investigate *A. thaliana* mutants defective in TRAPPC8, the only subunit that is both specific for this complex and has a homolog in yeast. We surveyed 2 T-DNA insertion lines with mutations mapped in *TRAPPC8*: SALK_124093 and SALK_130580.

Segregation of the mutant alleles did not follow the 1:2:1 pattern of inheritance (Table 1). For both mutants, an analysis of 48 plants derived from parental heterozygous lines showed that significantly less homozygotes and less heterozygotes were obtained, despite the fact that the parental lines showed no morphological abnormalities. We then crossed manually the heterozygous lines with wild-type (WT) plants, to see which parent displayed the defects (Table 1). The results were similar for both alleles: when pollen was derived from WT plants, segregation followed the Mendelian inheritance pattern, but when pollen was derived from heterozygous plants, disproportionately few mutant plants were obtained, defining a clear male transmission defect for *trappc8* mutations.

**Table 1.** Segregation of the *trappc8-1* and *trappc8-2* alleles

Cross	Expected ratio for Mendelian segregation	Observed ratio	Number of observations	*P*-value	Significance^c^
**Self-pollination**	WT:het:hom	WT:het:hom			
*trappc8-1^+/−^*	1 : 2 : 1	15 : 31 : 2	48	0.0038^a^	**
*trappc8-2^+/−^*	1 : 2 : 1	21 : 25 : 2	48	0.0005^a^	***
**Manual crossing**	WT:het	WT:het			
*trappc8-1^+/−^* ♀ × WT ♂	1 : 1	71 : 58	129	0.2541^b^	NS
WT ♀ × *trappc8-1^+/−^* ♂	1 : 1	118 : 10	128	<0.0001^b^	****
*trappc8-2^+/−^* ♀ × WT ♂	1 : 1	67 : 65	132	0.8624^b^	NS
WT ♀ × *trappc8-2^+/−^* ♂	1 : 1	90 : 9	99	<0.0001^b^	****

^a^Analyzed with the χ2 test against the H_0_ hypothesis that segregation is Mendelian.

^b^Analyzed with the binomial 2-tailed test against the H_0_ hypothesis that segregation is Mendelian.

^c^Significance: ****stands for *P*-value <0.0001, ***for *P* < 0.001, **for *P* < 0.01, NS - non-significant.

Still, in both cases, we were able to isolate homozygous lines; the location of the insertion sites was confirmed by PCR and sequencing (Supplementary Fig. S3, A and B). Both mutants produce truncated transcripts of the gene, but lack the full-length version (Supplementary Fig. S3, C and D). We termed them *trappc8-1* (SALK_124093, carrying an insertion in intron 20) and *trappc8-2* (SALK_130580, insertion in intron 10; this is the same mutant described before as *dqc-3* by Song et al. 2020 and as *trs85-2* by Allen et al. 2024).

**Figure 2.**
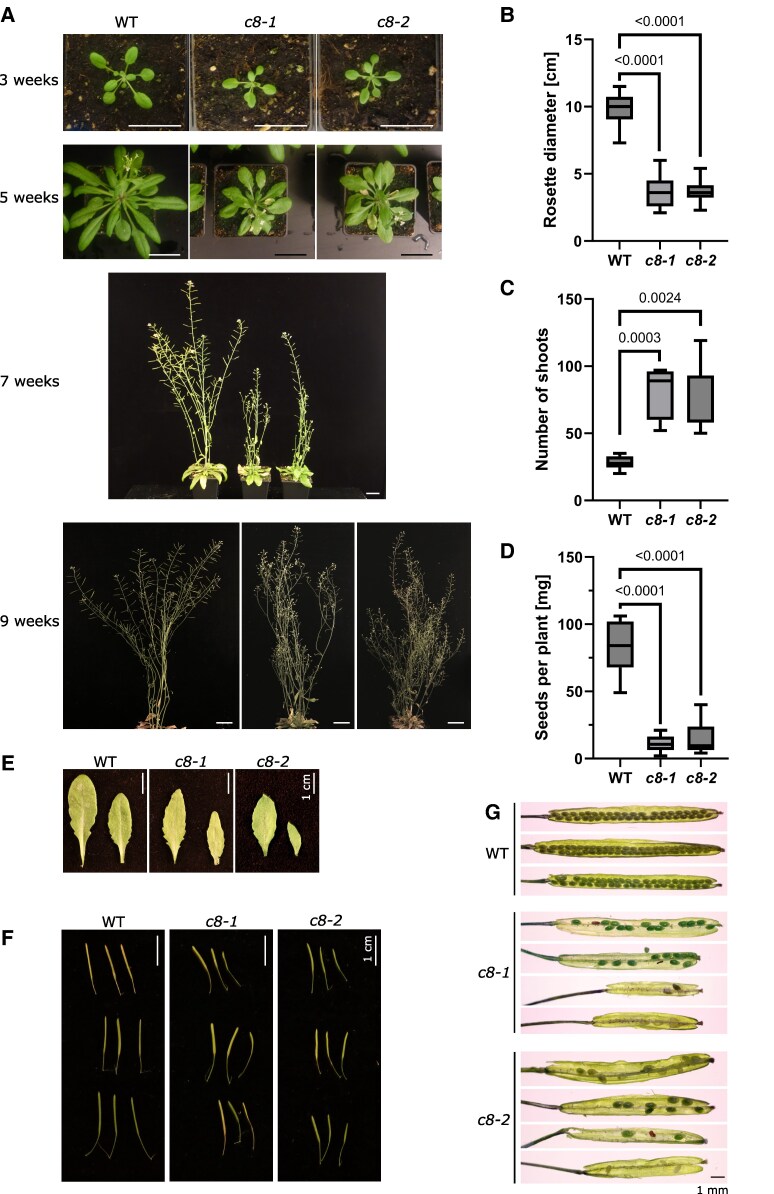
Phenotypic analysis of the mutant lines *trappc8-1* and *trappc8-2*. **A)** Overall morphology of WT and mutant plants cultured in soil. Scale bars: 3 cm. **B)** Quantification of rosette size of 5-wk-old plants. 20 plants were measured for each genotype. For **B–D)**: The box-and-whiskers plots show min to max values with median indicated and with whiskers representing the 25th and 75th percentile, *P*-values of unpaired *t*-tests with Welch's correction are indicated on the graphs. **C)** Quantification of total number of shoots (primary and secondary) of 9-wk-old plants. —Six to 7 plants were scored for each genotype. **D)** Quantification of amount of seeds produced per plant. The seeds of 8 to 14 individual plants were weighed for each genotype. **E)** Representative rosette leaves from 7.5-wk-old plants of the indicated genotypes. **F)** For 3 representative plants each, 3 mature but unopened siliques were photographed. **G)** The siliques shown in panel **F** were opened and photographed, representative examples are shown. The 1-mm scale bar is applicable to all images in panel **G)**. WT, wild-type; *c8-1*, *trappc8-1*; *c8-2*, *trappc8-2*.

**Figure 3.**
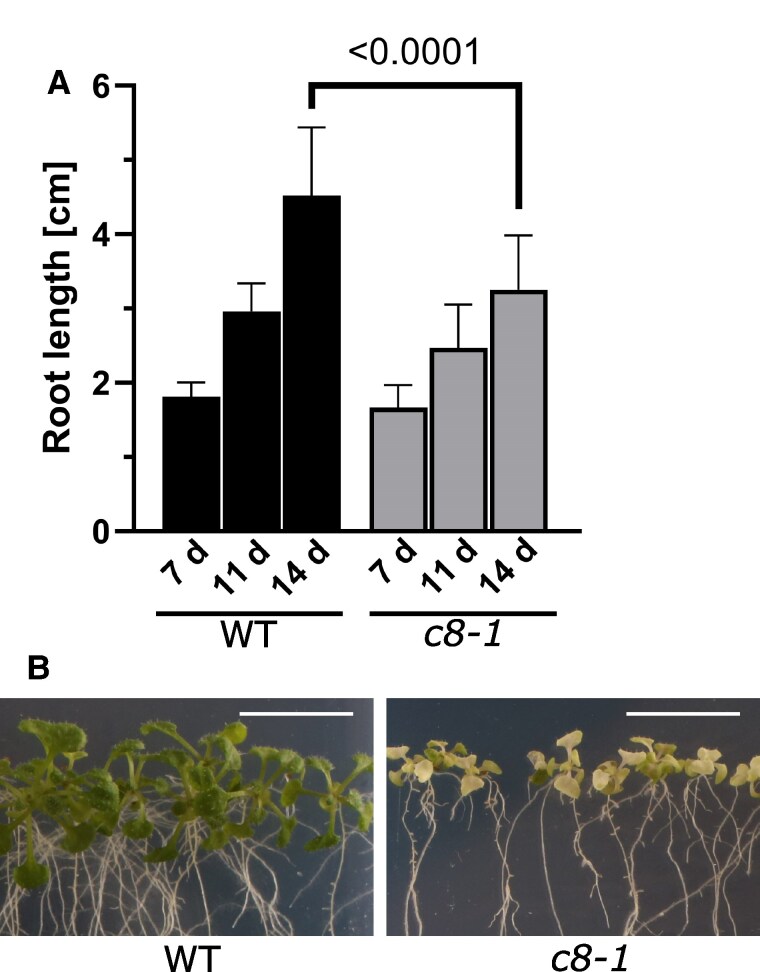
Phenotypes of *trappc8-1* mutant seedlings cultured on agar plates. **A)** Seedlings were cultured on vertical agar plates (1/2 MS) and photographed at 7, 11, and 14 d after sowing. Root length was measured using ImageJ. In total, 39 to 40 seedlings were measured for each time point. Mean values and standard deviations are shown. The *P*-value of an unpaired *t*-test with Welch's correction is indicated on the graph. **B)** After 21 d of growth, mutant seedlings show premature leaf bleaching. Scale bars: 1 cm. WT, wild-type; *c8-1*, *trappc8-1*.

Next, we characterized the overall morphology of both mutant plant lines. Data published previously on the *trappc8-2/dqc-3* line included only observations of root phenotypes in seedlings (Song et al. 2020), here, we looked also at entire plants. Both *trappc8* lines grew slower and were smaller than WT plants, they had smaller rosettes with pointed, more serrated leaves, kinked stem growth, and an excessive secondary branching phenotype (Fig. 2, A to C and E; for stem growth, see Fig. 5A below). Bolting time remained unchanged, but the flowering stage and silique development were prolonged. The plants produced small siliques (Fig. 2F), which were partially or completely empty (Fig. 2G). The mutants, though not completely sterile, produced very low amounts of seeds per plant (Fig. 2D). When cultured on plates, mutant seedlings showed slower root growth as well as premature leaf bleaching (Fig. 3). A mutant line carrying both analyzed *trappc8* alleles (*trappc8-1*/*trappc8-2*) displayed similar morphological phenotypes, confirming that the lines are allelic (Supplementary Fig. S4).

These morphological phenotypes remain in contrast to the appearance of the published *trappc11* mutants (Rosquete et al. 2019b), which resemble WT plants. We also directly compared the growth of *trappc8* and *trappc11* plants (Supplementary Fig. S5) and confirmed the major morphological differences. These results suggest that the TRAPPC8 subunit may perform functions additional to those performed together with TRAPPC11, as part of the TRAPPIII complex.

### TRAPPC8 is necessary for the development of seed coat cells and for mucilage deposition

**Figure 4.**
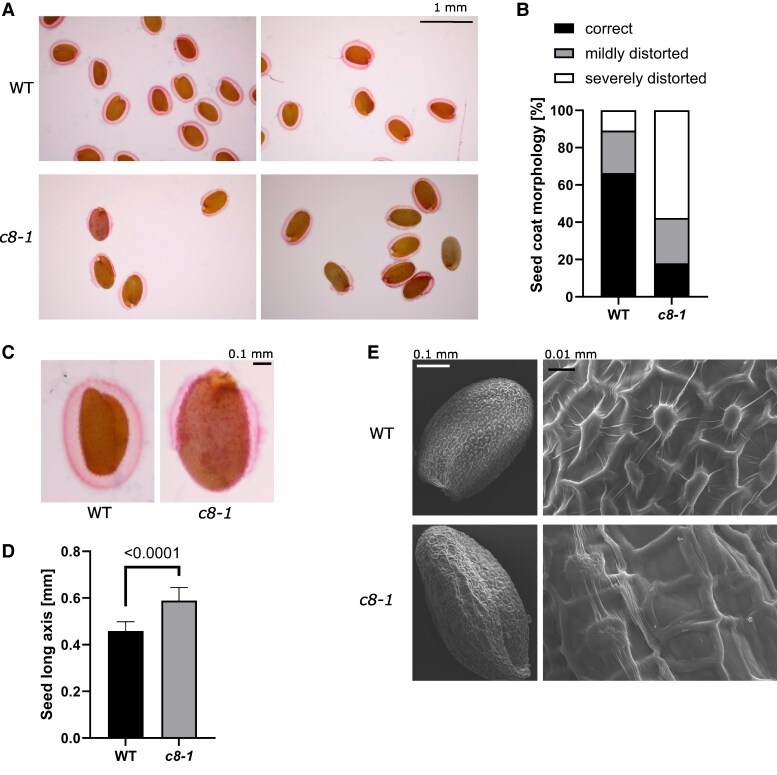
*trappc8-1* mutant plants produce abnormal seeds. **A)** WT and *trappc8-1* seeds were imbibed, stained with ruthenium red, and viewed under the microscope. Scale bar applies to all images in **A)**. **B)** Quantification of mucilage coat morphology. Approx. 80 seeds per line were manually assigned to 3 coat morphology categories: correct—the coat fully covers the seed, mildly distorted—the coat thins out/loses continuity in one spot, severely distorted—more parts of the seed are coatless. **C)** Close-up view of seeds from **A)**. Scale bar applies to both images in **C)**. Mutant seeds are larger and misshaped. **D)** Quantification of seed size. The same seeds as scored in **B** were measured (length of long axis) using ImageJ. Mean values and standard deviations are shown. The *P*-value of an unpaired *t*-test with Welch's correction is indicated on the graph. **E)** SEM images of WT and *trappc8-1* seeds. Scale bars apply to both upper and lower images. Aberrant morphology of the seed coat epidermal cells is visible. WT, wild-type; *c8-1*, *trappc8-1*.

We next inspected microscopically the seeds obtained from the homozygotic *trappc8* mutants. We observed imbibed seeds using ruthenium red staining, which visualizes acid polysaccharides. This method allowed us to assess not only seed size and shape, but also the morphology of the mucilage layers deposited on the seed outer surface. Seed morphology was visibly changed. The mutant seeds were larger and more elongated and had thinner mucilage layers (Fig. 4, A to D). The mucilage layers were often severely distorted, with large parts being very thin or completely absent. We visualized the seed outer surface by scanning electron microscopy (SEM), and we could see that the morphology of seed coat epidermal cells was defective (Fig. 4E). The epidermal cells were irregular and had distorted columellae, suggesting defects in the deposition of the secondary cell wall. Despite the observed defects, the seeds were viable: when sowed on plates, they germinated only slightly worse than WT seeds (92% compared with 99%). The observed seed phenotypes are similar to those noted previously for exocyst mutants (Kulich et al. 2010) and are consistent with the involvement of TRAPPIII in secretory processes.

### The trappc8 mutant has impaired flower development and pollen functioning

**Figure 5.**
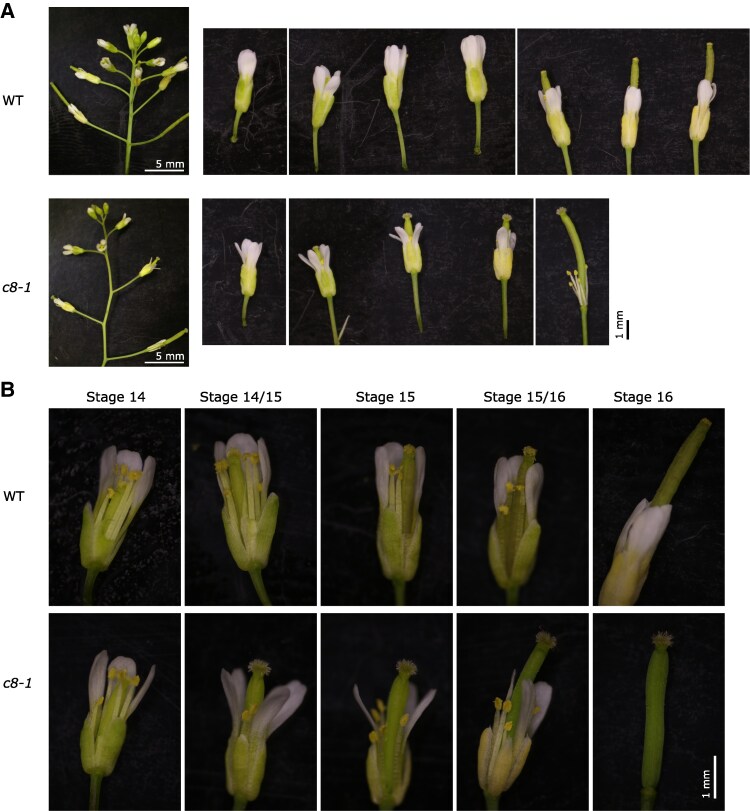
*trappc8-1* mutant plants have defective flower development. **A)** A representative inflorescence is shown for WT and *trappc8-1* plants. Note the kinked stem growth of the mutant. To the right, all flowers of the particular inflorescences are shown, starting from the first flower with visible petals. The 1-mm scale bar applies to all images of individual flowers. The protruding pistil of the mutant flowers is visible. **B)** Close-up of flowers from panel **A**. The 1-mm scale bar applies to all images in this panel. Flower development stages as defined by Smyth et al. (1990) are indicated. WT, wild-type, *c8-1*, *trappc8-1*.

The defective siliques prompted us to inspect the flowers of the *trappc8* mutant. We noticed that although initial stages of flower development proceed normally (up to stage 13 (Smyth et al. 1990)), later the pistil outpaces the stamens and petals (Fig. 5). This leads to a situation where the mature stigma of stage 14—which is functional in the mutants, as shown by the successful manual pollination (Table 1)—remains without contact with the dehiscing anthers and receives very little pollen, or none at all, and eventually dries out. This effect could account for the very low seed yield of the homozygous mutants, but the observed allele frequencies in the progeny of *trappc8* heterozygous lines (Table 1) led us to investigate also pollen functioning.

**Figure 6.**
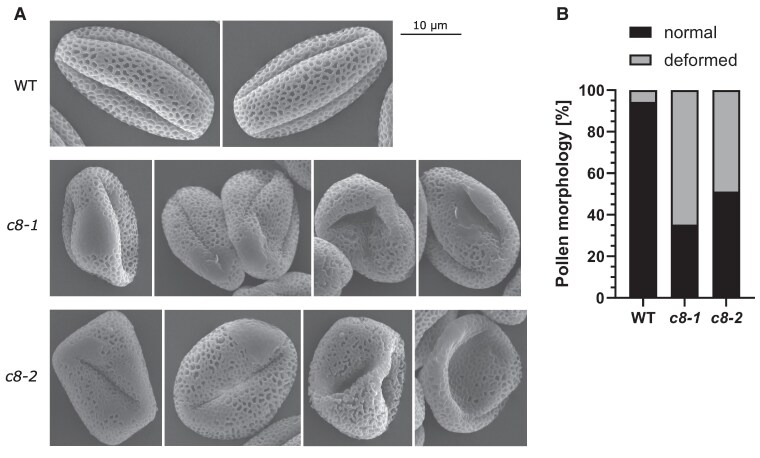
*Trappc8* mutant flowers produce aberrant pollen grains. **A)** SEM images of WT and *trappc8* pollen grains. The scale bar applies to all images in this panel. **B)** Quantification of photographed pollen. In total, 130 to 170 grains were scored per genotype.

Indeed, when we observed pollen grains by SEM, we found that they displayed visibly altered morphology (Fig. 6). Overall, the morphology of the grains was impaired, with about half of the grains misshaped. The structure of the pollen grain surface was also changed: the pollen cell wall structure was disturbed, in some regions of the grains even completely unformed. During pollen development, the pollen cell wall components are synthesized and secreted by tapetum cells (Ma et al. 2021), so the process is dependent on the correct functioning of the secretory pathway in these cells.

**Figure 7.**
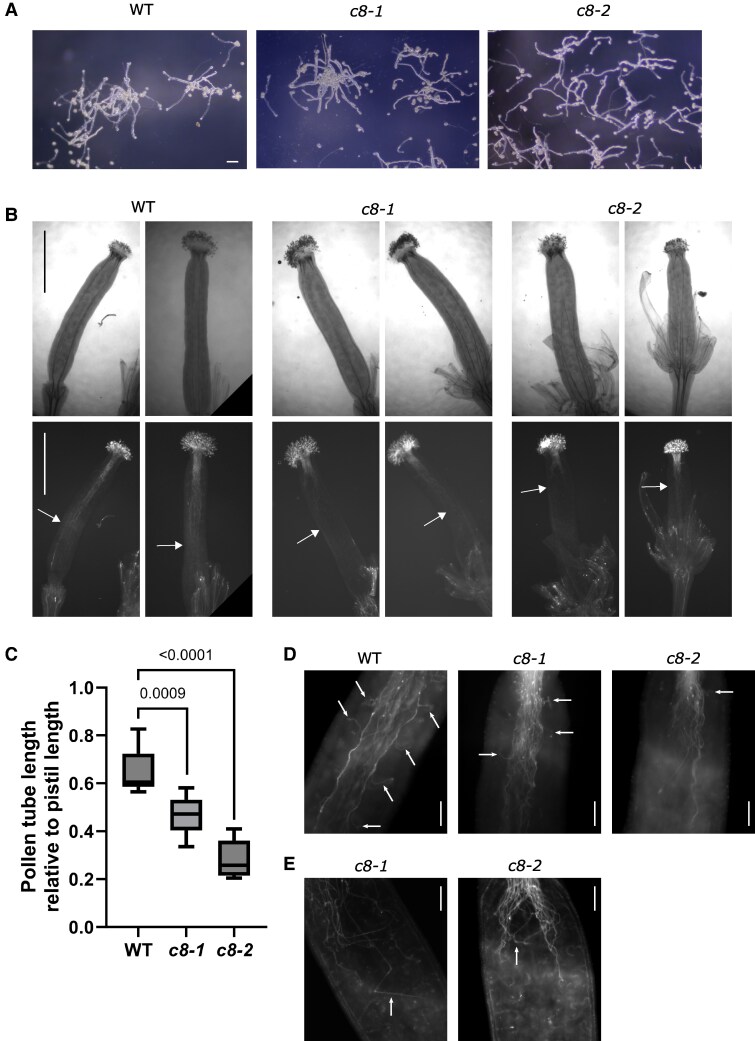
*Trappc8* pollen tubes are functionally deficient. **A)** WT and mutant pollen grains were germinated in vitro and photographed. Scale bars: 0.1 mm, applies to all images in this panel. **B)** WT and mutant pollen grains were placed manually on emasculated WT pistils, allowed to germinate for 10 h, fixed, stained with aniline blue, and viewed under the microscope. Arrows mark the furthest reaching pollen tube for each pistil shown. Scale bars: 1 mm, apply to all images in this panel. **C)** Quantification of experiment shown in **B)**. For each line, 8 to 9 pistils were scored. Min to max values are shown with median indicated and with whiskers representing the 25th and 75th percentile. *P*-values of unpaired *t-*tests with Welch's correction are indicated on the graph. **D)** Close-up views of representative stained pistils. Arrows mark instances where a pollen tube can be seen turning toward an ovary. Scale bars: 0.1 mm. **E)** Examples of mutant pollen tubes growing chaotically inside the pistils are marked with arrows. Scale bars: 0.1 mm. WT, wild-type; *c8-1*, *trappc8-1*; *c8-2*, *trappc8-2*.

We also reasoned that secretory pathway disturbances could lead to problems with pollen tube growth, which is highly dependent on secretion. However, when *trappc8* pollen grains were germinated in vitro, they were capable of growing tubes morphologically similar to those of WT pollen (Fig. 7A). We next performed hand-pollination of emasculated WT flowers to observe the germination of *trappc8* pollen grains on WT pistils. Here, we could clearly see that mutant pollen was able to adhere to the stigma and to germinate, and that the pollen tubes successfully made their way through the style (Fig. 7B), but did not reach as far down the transmitting tract as WT pollen tubes after 10 h (Fig. 7C), showing that they grow slower. Incidents where we could see a mutant pollen tube that left the transmitting tract and directed its growth toward an ovule were seldom when compared with WT pollen tubes (Fig. 7D). In some cases, the growth of the mutant pollen tubes inside the pistil seemed chaotic (Fig. 7E). These observations showed that the defect in secretion in the *trappc8* mutants is not so strong that it would prevent pollen tube growth (although it may be slowing it down), but rather that the ability of *trappc8* pollen to respond to signals emitted by the female gametophytes might be impaired. In particular, defects seem to occur at the stage of funicular guidance (Lopes et al. 2019), since the pollen tubes do not efficiently turn from the transmitting tracts toward the ovules.

The low fertility of the *trappc8* lines probably arises as a sum of the observed defects: the aberrant flower morphology (discrepancy between pistil and stamen size), disturbed pollen biogenesis, and impaired ovule-targeting competence of the male gametophyte.

### Arabidopsis TRAPPC8 is involved in autophagy

**Figure 8.**
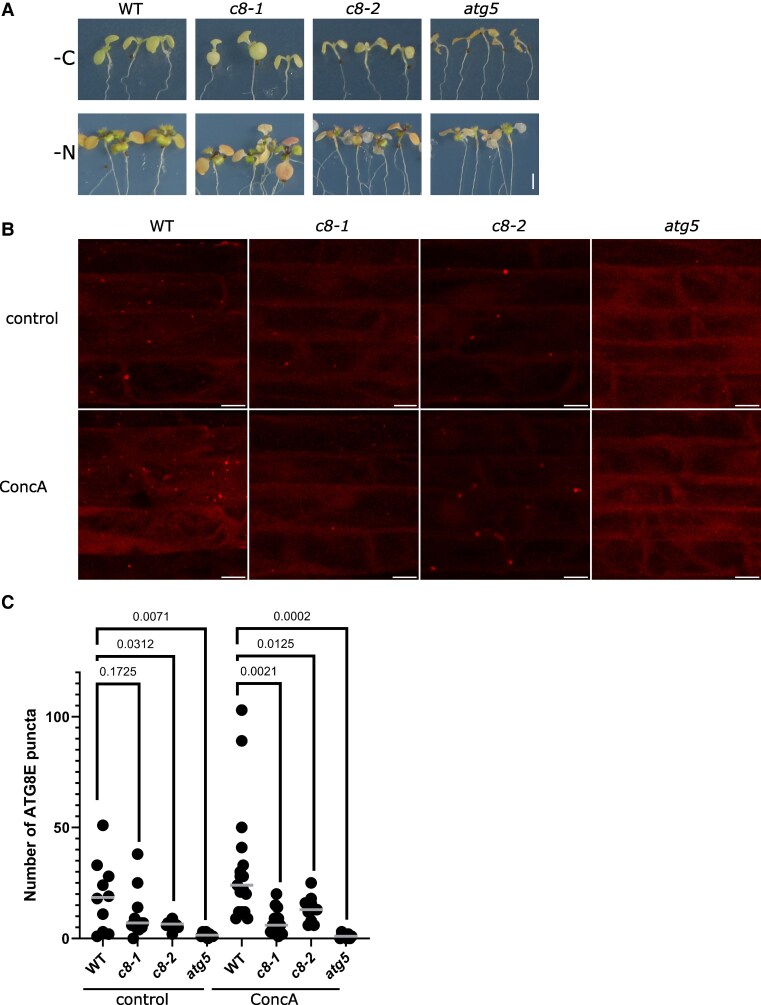
*trappc8* mutants display mild autophagy defects. **A)** 7-day-old seedlings of the indicated genotypes were transferred to conditions of either carbon (–C) or nitrogen (–N) starvation and observed for leaf chlorosis. Scale bar: 2 mm, applies to all images in this panel. **B)** Five-day-old seedlings expressing the fusion protein mCherry-ATG8E in the indicated genetic backgrounds were incubated for 2.5 to 3 h with or without ConcA and root epidermal cells were photographed in a confocal microscope with fixed settings. Representative images are shown. The image stacks were flattened for presentation. Scale bars: 10 *µ*m. **C)** mCherry-stained puncta were counted manually in the image stacks. The horizontal bars indicate the median for each result set. The numerical values on the graph are *P*-values for unpaired *t*-tests with Welch's correction. WT, wild-type; *c8-1*, *trappc8-1*; *c8-2*, *trappc8-2*; ConcA, Concanamycin A.

The yeast homolog of TRAPPC8, Trs85p, is an established factor in autophagy (Meiling-Wesse et al. 2005; Nazarko et al. 2005), and we hypothesized that Arabidopsis TRAPPC8 might also play a role in this process. To test this, we first assayed the starvation sensitivity of the mutants, since defects in autophagy typically lead to worse survival under starvation conditions. For all autophagy-related experiments, we used an *atg5* mutant line as a positive control: the AUTOPHAGY 5 (ATG5) protein is involved in the lipidation of ATG8 and is indispensable for autophagosome formation. We compared the growth of *trappc8* mutant seedlings with that of WT and autophagy-deficient *atg5* seedlings under conditions of nitrogen and carbon starvation. Judged by the extent of leaf chlorosis occurring, the *trappc8* mutants were slightly sensitive to nitrogen starvation, though not to carbon starvation (Fig. 8A, Supplementary Fig. S6A). However, the effect was very mild and should be viewed in the context of the phenotype of premature leaf chlorosis displayed by the *trappc8* mutants (see Fig. 3), so we looked for further data. Another general feature of autophagy-deficient plant mutants is a mild increase in total protein content per fresh weight (Guiboileau et al. 2013), resulting from defects in the turnover of protein aggregates. The *trappc8* mutants did show a slight increase in protein content, but in our hands even the control line *atg5* showed such a minor increase that this phenotype was difficult to assess (Supplementary Fig. S6B). We therefore further tested autophagic degradation also biochemically, by assaying the level of NEXT TO BRCA1 GENE 1 (NBR1), a cargo receptor for selective autophagy in plant cells (Zhang and Chen 2020). In *trappc8* seedlings, as in *atg5* seedlings, NBR1 protein content was strongly elevated in comparison to WT levels; however, the corresponding mRNA was also induced, ∼2-fold, potentially accounting partly for the protein accumulation. This result was consistent with an autophagy defect in the mutants, but again inconclusive (Supplementary Fig. S7).

Finally, to monitor autophagic flux directly, we constructed *trappc8-1* and *-2* lines expressing a fluorescent version of ATG8E (P_UBI_::mCherry-ATG8E). The ATG8E protein remains associated with autophagosomes from the stage of pre-autophagosomal membrane expansion until full autophagosome maturation and vacuole delivery, and it is an established autophagosome marker in Arabidopsis cells (Contento et al. 2005). Expression of the fusion protein was confirmed by Western blotting with an anti-mCherry antibody (Supplementary Fig. S8). Since the mutants showed only minor starvation sensitivity but a large increase in NBR1 levels under conditions that were not autophagy-inducing, we decided to assay basal autophagy. The notion that basal autophagy would be impaired in our mutants was also consistent with previous results from yeast cells, where TRAPPIII was found necessary for autophagy under nutrient-rich conditions (Shirahama-Noda et al. 2013). Five-day-old seedlings expressing the *mCherry-ATG8E* transgene in WT, *trappc8,* or *atg5* backgrounds were incubated with or without Concanamycin A (ConcA)—an inhibitor of the vacuolar H^+^-pump, which prevents proper acidification of the vacuole and thus the degradation of autophagosomes in its lumen—and then observed by confocal microscopy to assess the number of autophagosomes per given region in root epiderm (Fig. 8, B and C). Both *trappc8* mutants showed decreased numbers of autophagosomes compared with WT seedlings, confirming that autophagy is not proceeding properly in these cells. At the same time, in the presence of ConcA, there was a difference in phenotype penetration observable between the *atg5* and the *trappc8* mutants: in *atg5* cells, there were almost no ATG8E-positive dots at all, while in *trappc8* cells, some puncta were observed. This suggests that TRAPPC8 might play a regulatory role in autophagy rather than being structurally indispensable for the process.

### Induction of the UPR in trappc8 mutants

**Figure 9.**
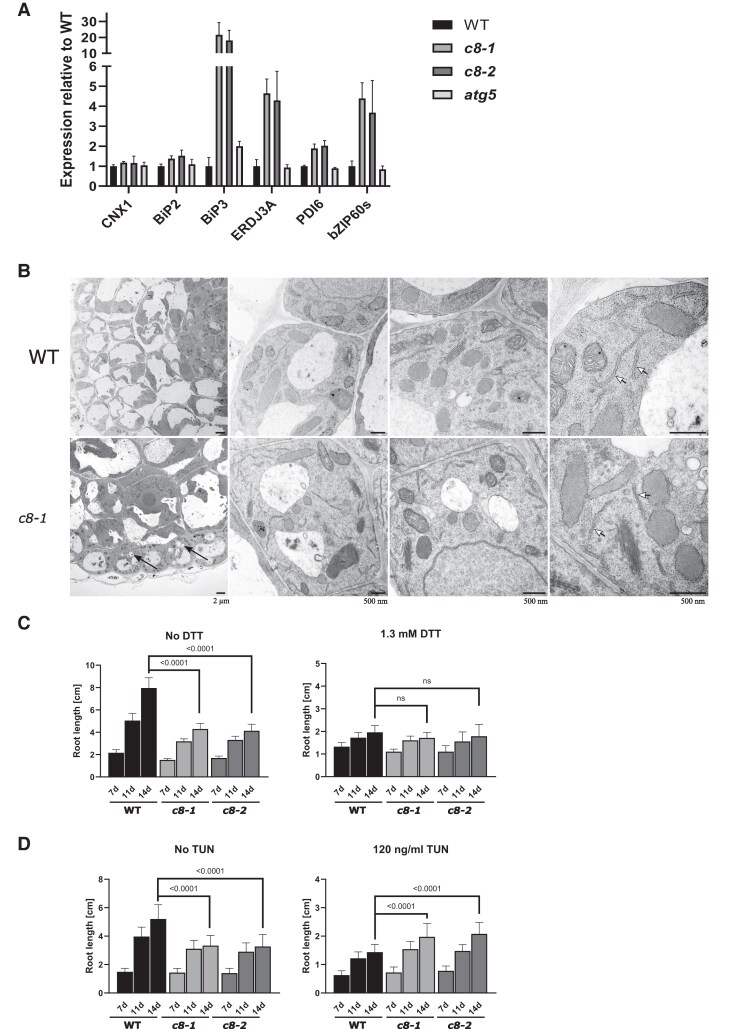
*trappc8* mutants display a constitutive ER stress response and changed sensitivity to ER stress agents, but no changes in ER morphology. **A)** RNA extracted from seedlings of the indicated genotypes grown for 2 weeks in a liquid medium was used for RT-qPCR with primers detecting 6 UPR marker transcripts. Three biological replicates were performed, mean values and standard deviations are shown. **B)** Seven-day-old seedlings cultured on plates were fixed and root sections were visualized by TEM. Representative images at increasing magnification are shown. Black arrows point to examples of small, rounded vacuoles visible in the mutant cells (lower leftmost panel), white arrowheads point to ER cisternae (rightmost panels). Scale bars: 2 *µ*m for leftmost panels, 500 nm for other panels. **C and D)** WT and *trappc8* seeds were sowed on plates containing the indicated concentrations of DTT or TUN in the growth medium. Seedlings (22 to 32 for the DTT experiment, 28 to 30 seedlings per genotype for the TUN experiment) were photographed after 7, 11, and 14 days of growth, and root length was measured using ImageJ. Mean values with standard deviations are shown. The values given on the graphs are *P*-values for unpaired *t*-tests with Welch's correction; ns, nonsignificant (*P*-value above 0.05). WT, wild-type, *c8-1*, *trappc8-1*, *c8-2*, *trappc8-2*.

Defects in protein trafficking through the early secretory pathway may cause protein accumulation in the ER and lead to ER stress (Pastor-Cantizano et al. 2018). If TRAPPIII would be required for trafficking at steps earlier than TGN/EE compartments, such as ER exit and ER-to-Golgi trafficking, then *trappc8* mutations could cause ER stress and lead to induction of the unfolded protein response (UPR). To investigate this, we assayed the *trappc8* mutants for the transcriptional upregulation of several UPR markers. These included the ER chaperones: CNX1 (CALNEXIN 1, recognizes misfolded transmembrane and glycosylated proteins (Bloemeke et al. 2022, and references therein)), BiP2 and BiP3 (LUMINAL BINDING PROTEIN 2 and 3, chaperones for soluble ER lumen proteins) and their J-domain interactor ERDJ3A (ER-LOCALIZED DNAJ PROTEIN 3A (Pobre et al. 2019)), and the protein disulfide isomerase PDI6 (PROTEIN DISULFIDE ISOMERASE 6, also known to participate in recognizing misfolded clients in the ER lumen (Lu and Christopher 2008)). We also included analysis of the bZIP60s transcript—an unconventionally spliced version of the mRNA for the transcription factor BASIC REGION/LEUCINE ZIPPER MOTIF 60 (bZIP60)—which is induced by UPR and by INOSITOL-REQUIRING 1 (IRE1) activation (Nagashima et al. 2011). All of these are established markers of the UPR in *A. thaliana* (Pastor-Cantizano et al. 2018). As shown in Fig. 9A, transcriptional induction was very strong for BiP3 and ERDJ3A; for PDI6, a mild increase was observed. Also, the bZIP60s mRNA was strongly induced. Together, these results show that the *trappc8* mutants have constitutive activation of the UPR.

To rule out the possibility that ER stress in the analyzed mutants results from the observed defects in autophagy, which could impair the cell's ability to clear protein aggregates accumulating in the ER, we included the *atg5* mutant in the analysis (Fig. 9A). The mutant did not display any significant UPR induction, which was consistent with previous data for the *A. thaliana atg5* mutant (in a transcriptomics analysis, no induction was found for UPR genes; Havé et al. 2019) as well as with data from yeast cells (mutations impairing autophagy did not lead to an induction of the UPR unless they were accompanied by an overproduction of ER proteins; Lipatova et al. 2020). This suggests that the constitutive ER stress observed in *trappc8* is not a secondary effect of autophagy deficiency, but results rather from direct defects in ER functioning.

To see if the UPR induction is accompanied by changes in ER morphology, we performed transmission electron microscopy (TEM) imaging of root cells from WT and *trappc8-1* seedlings. We visualized epidermal and cortical cells in the root meristematic growth zone (Fig. 9B). The previously reported fragmentation of vacuoles in root epiderm (Song et al. 2020) could be observed in some cells (Fig. 9B). However, as judged from the micrographs, the abundance and morphology of ER cisternae, as well as that of Golgi structures, were unchanged in the mutant.

We then used a plate growth assay to investigate the sensitivity of *trappc8* seedlings to 2 ER stress agents: dithiothreitol (DTT; causes protein stress by disrupting disulfide bonds) and tunicamycin (TUN; causes stress by blocking the enzyme UDP-GlcNAc-1-P: DolP transferase and disrupting protein glycosylation), to see if the UPR activation enables the cells to better cope with these types of stress. The mutants showed a mild root growth defect when cultured on regular media (Fig. 3A, Fig. 9, C and D); in the presence of 1.3 mm DTT, the mutant grew at a rate similar to that of WT seedlings, showing decreased sensitivity (Fig. 9C; additional data Supplementary Fig. S9A). Interestingly, when ER stress was elicited by the addition of 120 ng/ml TUN, the mutant seedlings were markedly less affected than WT seedlings and had significantly longer roots than WT seedlings (Fig. 9D; data for lower concentrations of the compounds in Supplementary Fig. S9B). Thus, to our surprise, the loss of full-length TRAPPC8 actually conferred TUN resistance. We conclude that *trappc8* plants display alterations in ER functioning that result in constitutive activation of the UPR, leading to more flexibility in adaptation to ER stress, especially glycosylation stress. This observation prompted us to directly analyze protein glycosylation and Dol levels in *trappc8* lines.

### Effects of TRAPPC8 loss on protein glycosylation and Dol levels

The established function of TRAPPIII is as a GEF for Rab1/Ypt1 in animal and yeast cells (Galindo et al. 2021; Harris et al. 2021; Joiner et al. 2021). However, in human patients, *trappc11* mutations are associated with a range of glycosylation disorders (Corona-Rivera et al. 2024). In zebrafish embryos and HeLa cells, mutation of the *TRAPPC11* subunit gene has been shown to cause defects in glycosylation of a marker protein and lowered levels of lipid-linked oligosaccharide (LLO) precursors and was synthetically lethal with treatment with TUN (DeRossi et al. 2016). In that work, the LLO decrease was not visible after siRNA-silencing of the gene for the TRAPPC8 subunit, but the expression of the targeted protein was only partially suppressed under the applied experimental conditions. We reasoned that some of the phenotypic traits described here would be consistent with a potential defect in protein glycosylation: defects in communication between the pollen tube and female gametophyte (Fig. 7) have been previously described for *N*-glycosylation-deficient mutants (Lindner et al. 2015) and for glycosylphosphatidylinositol (GPI)-anchoring-deficient mutants (Capron et al. 2008), and the flower and pollen grain morphology of *trappc8* plants (Figs. 5 and 6) resembled that of *pprd2* mutants, which are deficient in the biosynthesis of Dol, an indispensable cofactor of glycosylation (Jozwiak et al. 2015); however, the TUN resistance of *trappc8* seedlings suggested otherwise. We decided to look directly at protein glycosylation and Dol levels in *trappc8* plants.

**Figure 10.**
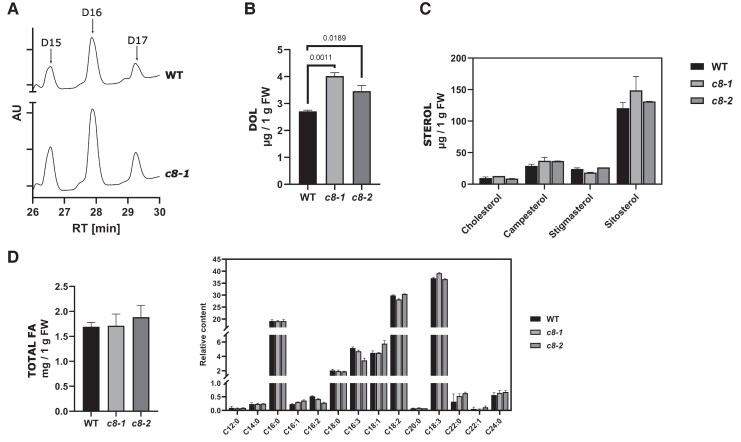
*trappc8* mutant plants show increased accumulation of Dol but not other lipids. The indicated lipids were isolated from WT and mutant seedlings grown for 2 weeks in a liquid medium. **A)** The samples were analyzed by HPLC/UV. Representative chromatograms are shown, with peaks corresponding to dolichol-15 (D15), dolichol-16 (D16), and dolichol-17 (D17) marked. AU, arbitrary units; RT, retention time. **B)** Quantification of Dol accumulation. The sum of Dol-15, -16, and -17 is plotted. **C)** Quantification of 4 main phytosterols. **D)** Total accumulation of fatty acids (FA) and relative content (percent of total) of identified FAs (indicated below the graph by chain length and saturation level). FW, fresh weight. For **B–D)**, 3 biological replicates were assayed in each experiment and mean values with standard deviations are shown. The numerical values on the graph in **B** are *P*-values for unpaired *t*-tests with Welch's correction. For sterols and fatty acids, no significant differences were found between WT and mutant seedlings. WT, wild-type; *c8-1*, *trappc8-1*; *c8-2*, *trappc8-2*.

First, we tested extracts from WT and mutant seedlings with antibodies against the specific protein SKEWED 5 (SKU5), which is known to be both strongly glycosylated and decorated with a GPI anchor and can thus serve as a marker of glycosylation defects in *A. thaliana* (Sedbrook et al. 2002), but neither a change in gel migration nor lowered levels of SKU5 were detected (Supplementary Fig. S10). We concluded that in the absence of TRAPPC8, protein glycosylation can be conducted efficiently. To address the TUN resistance of *trappc8* plants, we measured the levels of DolDol in seedlings cultured in a liquid medium. Both mutant lines showed an increase in Dol content up to 130% to 150% of WT values (Fig. 10, A and B). The results were similar in lyophilized seedlings, showing that they were not resulting from differences in water content (Supplementary Fig. S11A). The Dol content was elevated also in rosette leaves of 4-week-old plants cultured in soil, confirming that this was a mild but reproducible effect, not connected to culture conditions (Supplementary Fig. S11B). This result was surprising, but it was in line with the TUN resistance, since dolichol phosphate (Dol-P) is one of the substrates of the TUN target, UDP-GlcNAc-1-P­: Dol-P ­­transferase. To put this result in context with other ER lipids, we measured the levels of the 4 main phytosterols and of fatty acids; the levels of these lipids did not change significantly (Fig. 10, C and D), suggesting that the lipid composition of the ER membrane is not strongly distorted.

To find out if the enhancement of the Dol accumulation results from an induction of its biosynthesis, we checked if the transcription of enzymes responsible for Dol biosynthesis was upregulated in the mutants (Supplementary Fig. S12). The transcription of the genes implicated in Dol biosynthesis through the mevalonate (MVA) and methylerythritol (MEP) pathways (Lipko et al. 2023): *HMGR1*, *HMGR2*, *MVK*, *DXS*, *DXR*, *MCT*, as well as of 2 FPP synthase genes: *FPPS1* and *FPPS2*, remained at WT levels. The genes for the 2 final enzymes of Dol biosynthesis: *CPT3* (Gawarecka et al. 2022) and *PPRD2* (Jozwiak et al. 2015), and especially the gene *EVN,* which encodes a Dol kinase (Lindner et al. 2015), showed a minor, but nonsignificant, increasing trend. These results suggest that the rate of Dol biosynthesis is probably not increased in the *trappc8* mutants.

We considered then the possibility that elevated Dol levels result from reduced rates of Dol degradation, but no specific mechanism of Dol degradation in cells has been described yet. We reasoned that, since Dol resides in the ER membrane and the ER is likely an important source of membrane for autophagy (Zhen and Stenmark 2023), a decrease in autophagic membrane turnover could be the underlying cause of decreased Dol degradation. To test this idea, we measured Dol levels in several homozygous *atg* mutants of *A. thaliana*: *atg2*, *atg5*, *atg9*, *atg11*, *atg13*. However, these mutants, though autophagy-deficient, mostly showed no significant increase in Dol levels (Supplementary Fig. S13A); only *atg2* showed a mild accumulation (up to 130%) similar to the effect visible in *trappc8* lines. We also tested the effects of TUN on the growth of a representative *atg* mutant line (*atg5*) and we did not observe any TUN resistance effect (Supplementary Fig. S13B). These results show that the Dol increase observed in our mutants is specific for *trappc8*, not a general effect of autophagic deficiency.

## Discussion

In this study, we build on the known structure of the metazoan TRAPPIII complex to propose an initial structure of plant TRAPPIII. Both data on binary interactions among subunits and theoretical modeling show that the general architecture of the plant complex is similar to that of metazoan TRAPPIII. A comparison of plant and animal TRAPPIII structures did not reveal any differences with clear functional significance, underscoring the high conservation of the complex. To learn more about the functions of plant TRAPPIII, we characterized one of its key subunits, TRAPPC8, by performing a detailed analysis of mutant plants.

### TRAPPC8 and the regulation of membrane traffic

Previous work has shown that aberrant protein sorting in *trappc8* root cells causes the auxin efflux carrier PIN1 to be partly mislocalized to the vacuole, leading to an aberrant auxin response, which was associated with defects in root growth and gravitropism (Song et al. 2020). More recently, defects in both exo- and endocytosis of cellulose synthase complexes have been demonstrated in *trappc8* seedlings (Allen et al. 2024), leading to defective growth of etiolated seedlings. The developmental defects observed by us can also be largely linked to misfunctions in protein sorting and secretion: small size of plants, excessive branching, serrated leaves, kinked stem growth, delayed flowering, and pronounced fertility defects resulting in small siliques. These phenotypes can be explained by disturbances in delivery to the plasma membrane of integral or secreted components, pointing to the role of TRAPPC8 in the secretory pathway and in component recycling.

Some auxin mutants display similar flowering and fertility phenotypes (Tan et al. 2023), and in particular, mutants in the gene *AUXIN RESPONSE FACTOR 2* (*ARF2*) have increased seed size and floral phenotypes very similar to those of *trappc8* (Schruff et al. 2006). Incorrect sorting and secretion of proteins may also underlie the changes in seed coat epidermal cells and in seed mucilage deposition (Kulich et al. 2010) as well as the abnormal morphology of *trappc8* pollen grains because efficient secretion of components by tapetum cells is necessary for the formation of the exine layer (Zhao et al. 2016; Aboulela et al. 2018). The defects in pollen tube guidance may be related both to impaired delivery of membrane-bound receptors (Guan et al. 2014; Stührwohldt et al. 2015) and to impaired ER functions (Wang et al. 2008; Lu et al. 2011).

The pollen grain cell wall defects were similar in *monensin sensitivity 1* (*mon1*) mutants (Cui et al. 2017), which are defective in the activation of RABG proteins. RABG proteins localize to vacuolar and prevacuolar membranes, where they regulate traffic coming both from the endocytic and autophagic routes (Kwon et al. 2013; Cui et al. 2017). Also, Rab prenylation mutants, which have partial defects at multiple inner trafficking routes, display similar abnormal flower morphology and pollen phenotypes (Gutkowska et al. 2015, 2021). Finally, the *trappc8* phenotypes strongly resemble those of *rabD2* mutants. The double mutant *rabD2b rabD2c* was described to have short siliques, defects in pollen grain morphology (with cell wall defects similar to those of *trappc8* pollen), decreased pollen tube growth rate, and a reduced seed set (Peng et al. 2011). Therefore, the phenotypic data for *trappc8* mutants supports the involvement of the protein in regulating membrane traffic at more than one site, potentially through Rab activation.

### TRAPPC8 and autophagy

We show that the process of autophagosome formation is impaired in *trappc8* cells. Under noninducing conditions, we saw a significant decrease in the number of autophagosomes being formed. At the same time, in induced (starved) cells overall autophagic flux was not dramatically low, as shown by the very mild starvation survival phenotypes of the mutants. These results are consistent with data from other organisms. In yeast cells, TRAPPIII was found necessary for Atg9p cycling from early endosomes to the ER under nutrient-rich conditions, while the requirement was by-passed upon starvation (Shirahama-Noda et al. 2013); also in mammalian cells, TRAPPIII subunits were necessary for ATG9A recycling and for autophagosome formation (Imai et al. 2016; Lamb et al. 2016) or for autophagic flux under selected conditions (Ramírez-Peinado et al. 2017; Stanga et al. 2019). In Arabidopsis, it was shown that the role of ATG9 for autophagosome formation is indispensable (Zhuang et al. 2017), and our data suggest that here, as in yeast, the requirement for TRAPPC8 might be by-passed under starvation conditions. Whether the role of TRAPPC8 in plant autophagy is connected to ATG9 recycling remains to be determined. Interestingly, in plants, ER stress is a known inducer of autophagy (Liu et al. 2012), but *trappc8* mutants, despite constitutive ER stress, do not undergo an induction of autophagosome formation, showing that this induction pathway—unlike starvation—probably requires TRAPPC8 function. Our results support, therefore, the concept that there are various routes in plant cells for autophagy signaling.

### TRAPPC8 and ER functioning

Some of our results point to a requirement for TRAPPC8 in ER homeostasis and functioning. First, *trappc8* mutants have constitutive activation of the UPR response, which could signal an accumulation of misfolded or otherwise aberrant proteins in the ER, and this effect is not secondary to their autophagy deficiency. Second, we find that overall levels of Dol, an ER lipid serving as a cofactor for protein glycosylation, are elevated in *trappc8* mutants, while other assayed ER lipids remain unchanged and no transcriptional induction in the Dol biosynthesis pathways can be observed. We reasoned that this might result from decreased Dol turnover, e.g. through a decreased rate of lipid flow into autophagosome membranes, but most tested *atg* mutants had normal Dol levels; however, the line deficient in the lipid transfer protein ATG2 showed a somewhat similar Dol phenotype.

The increase in Dol levels is (consistently) accompanied by TUN resistance of *trappc8* seedlings, and accordingly, protein glycosylation proceeds efficiently. This observation is in line with a recent report describing an Arabidopsis Golgi trafficking mutant (*CONSERVED OLIGOMERIC GOLGI COMPLEX 7*, *cog7*) which displayed enhanced *N*-glycosylation in the Golgi apparatus (elevated transcript levels of glycosyltransferase genes) (Choi et al. 2023), but it remains in contrast to observations from animal cells, where mutations in a different TRAPPIII subunit, TRAPPC11, led to decreased levels of LLOs and glycosylation defects (however, Dol levels were not tested in any animal models) (DeRossi et al. 2016; Matalonga et al. 2017; Larson et al. 2018; Munot et al. 2022; Corona-Rivera et al. 2024). We directly tested Dol levels in 2 *A. thaliana trappc11* mutants (Supplementary Fig. S14) and confirmed that in these lines, no decrease in Dol occurs (though no consistent increase either). The mechanism linking TRAPPC11 with protein hypoglycosylation in animal cells awaits explanation. It could reflect a function specific to the C11 subunit, or a function characteristic for the metazoan protein. In plants, *trappc11* mutants do not display the same morphological and fertility phenotypes as the *trappc8* lines, and also the function in cellulose synthase trafficking is specific for the TRAPPC8 (TRS85) subunit (Allen et al. 2024). The role in regulating Dol levels could also reflect a specific function: the biosynthesis of Dol in plants occurs by the interplay of 2 metabolic pathways, one of which is cytoplasmic and is common with animal cells (MVA pathway), while the other is located in plastids and does not have an animal counterpart (MEP). Both pathways contribute intermediates for Dol biosynthesis and both are likely subject to different regulatory mechanisms (Lipko et al. 2023). Together, this demonstrates that TRAPPIII must be subject to a complex regulatory system.

### TRAPPC8 and senescence

The premature senescence phenotype of *trappc8* mutant plants could be connected both to autophagy dysfunction and Dol accumulation. *atg* mutants display early leaf senescence in response to various nutrient-limiting conditions (reviewed by Chen et al. 2019). Increasing Dol levels have long been considered a marker of aging in animal tissues (Chojnacki and Dallner 1988; Parentini et al. 2005), and in plants, Dol levels are also known to increase with age (Gawarecka et al. 2022). Interestingly, the recent work of Choi et al. (2023) shows that a defect in the functioning of retrograde transport from the Golgi apparatus can be the primary cause of both premature leaf senescence and enhancement of the glycosylation machinery—in line with our results, which show similar effects in the context of an ER dysfunction.

### TRAPPIII and TRAPPII

The TRAPPIII and -II complexes share a 7-subunit core structure and have at least partially overlapping localization. The complex core is indispensable for GEF activity, but also the TRAPPIII and -II specific subunits are involved in efficient Rab binding and nucleotide exchange (reviewed in Sun and Sui 2023), and they determine the transition between GEF activity toward Rab proteins of different families, serving also a regulatory role in the complexes. When considering the cellular functions of TRAPPII and -III, the competition for the core subunits is an important aspect that has not been investigated yet. Recently, a role has been proposed for plant TRAPPII in the integration of stress signals (Wiese et al. 2024). Such a role in developmental decisions at the TGN is likely being executed in the interplay between the TRAPPII and -III complexes.

## Conclusions

**Figure 11.**
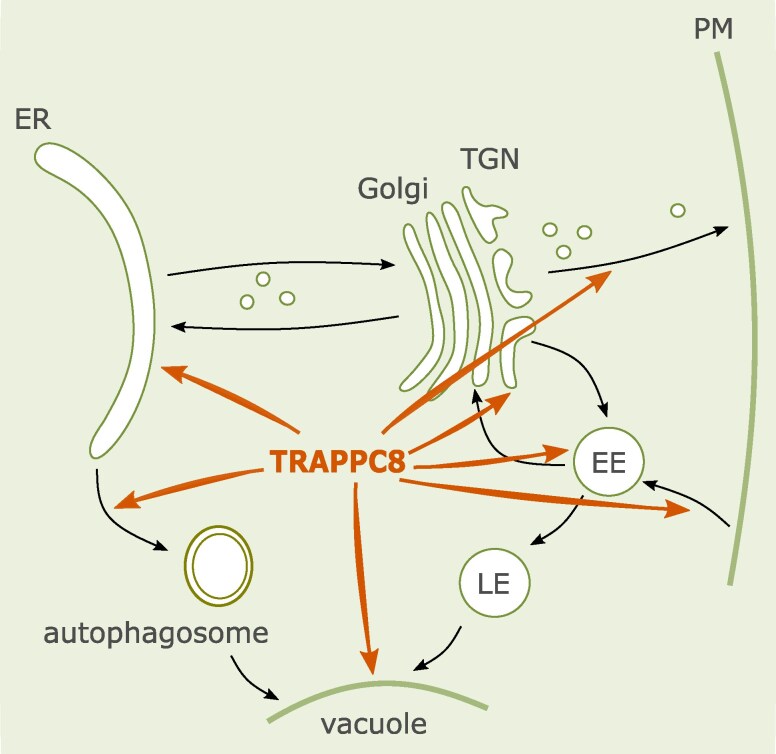
Compartments and trafficking steps influenced by TRAPPC8, a subunit of the TRAPPIII complex. The scheme is based on data from Song et al. (2020), Allen et al. (2024), and this study. ER, endoplasmic reticulum; TGN, *trans*-Golgi network; EE, early endosomes; LE, late endosomes; PM, plasma membrane. AtTRAPPC8 is necessary for correct protein sorting at the TGN and EE, secretion, endocytosis, regulation of transport to the vacuole, as well as for the biogenesis of autophagosomes and correct ER functioning (arrows).

Results described in this report document that the architecture of the TRAPPIII complex is conserved in Eukaryotes. TRAPPC8—either as part of TRAPPIII, or separately—is implicated in numerous events of intracellular trafficking, ranging from secretion and recycling to autophagy and ER functions (Fig. 11). This wide spectrum of cellular processes involving TRAPPC8 must result in a complex map of interactors—to be discovered yet. Interestingly, the misfunction of TRAPPC8 leads to increased accumulation of Dol, which in turn protects the cells against deficiencies in protein glycosylation.

## Materials and methods

### Plant lines and growth conditions

In this work, Arabidopsis plants (*A. thaliana*) of the Col-0 ecotype were used as the WT control line. The analyzed *trappc8* mutants were derived from lines obtained from the NASC collection: SALK_124093 (*trappc8-1*) and SALK_130580 (*trappc8-2*, previously described as *dqc-3* by Song et al. 2020, and as *trs85-2* by Allen et al. 2024)). Primers used for genotyping of the SALK lines are given in Supplementary Table S1. The same primers were also used to sequence the PCR products resulting from the genotyping procedure, which allowed us to determine precisely the localization of the T-DNA insertions in these lines. The double mutant *trappc8-1*/*trappc8-2* was obtained by manual crossing of heterozygous single mutants followed by PCR-based progeny analysis.

*trappc11* mutants were obtained from the NASC collection. The line referred to as *trappc11-6* was derived from SAIL_118F07 and *trappc11-7* was derived from WiscDSLoxHS012_05B (both previously described as *attrappc11/rog2-*6 and *attrappc11/rog2-7* by Rosquete et al. 2019b). The line *nbr1* was a gift from Dr Anna Wawrzyńska (IBB PAS, Warsaw, Poland; described as KO1 in Tarnowski et al. 2020). The line harboring an integrated transgene *pUBI::mCherry-ATG8E* in the WT background was a gift from Dr. Jiwen Liang (Chinese University of Hong Kong, Hong Kong, China; described in Hu et al. 2020), and the line harboring *P_UBI_::mCherry-ATG8E* in the *atg5* background, as well as the lines *atg5* itself (derived from SAIL_129_B07) and *atg2-2*, *atg9-3*, *atg11-1*, and *atg13* were described previously by Stephani et al. (2020). Lines *trappc8-1 [mCherry-ATG8E]* and *trappc8-2 [mCherry-ATG8E]* were obtained by crossing of the lines *trappc8-1* and *−2* (♀) to pollen from the line harboring *[mCherry-ATG8E]* (♂). All primers used for genotyping are listed in Supplementary Table S1.

For the greenhouse culture, plants were grown in soil under standard long day (16 h light/8 h dark) conditions at 22 °C. For plate assays, seeds were sown on ½ Murashige and Skoog (MS) medium supplemented with vitamins, solidified with 1.3% agar, stratified for 2 d, and grown vertically in a growth chamber at 22 °C, with 70% humidity and 16 h light/8 h dark period. Where indicated, tunicamycin (TUN, Sigma, T7765) was added to the medium from a 1 mg/ml stock in dimethyl sulfoxide (DMSO) to a final concentration of 80, 100, or 120 ng/ml. For the dithiothreitol (DTT) assay, DTT was added from a 2 m stock in DMSO to final concentrations of 0.7, 1.0, 1.3 mm, and 3-day-old seedlings were manually moved from regular plates to the DTT test plates. For biochemical experiments, seedlings were grown in liquid ½ MS medium supplemented with 1% sucrose for 2 wk on a rotary shaker. Conditions used for the starvation sensitivity assays and for confocal microscopy are given below.

### Plasmid construction

The *A. thaliana* TRAPP genes used in the yeast Y2H assay (encoding the subunits AtTRAPPC8 (At5g16280), −C11 (At5g65950), −C12 (At4g39820), −C13 (At2g47960), −C2 (At1g80500), −C2L (At2g20930)) were cloned from cDNA prepared either from leaves (−C8, −C13, −C2, −C2L) or flowers (−C11, −C12) of WT plants. The coding sequences were amplified by PCR with appropriate primer pairs (Supplementary Table S1) and cloned either into the pDONR201 vector by recombination, using the Gateway BP Clonase II Enzyme Mix (Invitrogen), or into the pENTR/d-topo vector (Invitrogen) according to the manufacturer's instructions. Correct orientation and nucleotide sequence of the products were verified by sequencing. Next, the DNA fragments were transferred by recombination into the appropriate yeast Y2H vectors, using the LR Clonase II Kit (Invitrogen). For N-terminal fusions with the GAL4-AD or -BD the vectors pGADT7-GW and pGBKT7-GW (gift from Yuhai Cui, Addgene plasmids #61702, 61703; (Lu et al. 2010)) were used, for C-terminal fusions we used pGADCg and pGBKCg (gift from Peter Uetz, Addgene plasmids #20161, 20162; (Stellberger et al. 2010)). All constructs were confirmed by sequencing. The *Escherichia coli* strain used for plasmid propagation was DH5α.

### Yeast Y2H assay

The yeast strain used as host for the Y2H assay was AH109 (James et al. 1996). The appropriate plasmids were transformed into yeast cells by the LiAc-PEG method (Chen et al. 1992), either consecutively or simultaneously. Apart from the test hybrid pairs, each construct was also cotransformed into yeast cells together with the appropriate empty vector (allowing for the expression of the GAL4-AD or –BD alone), to serve as a control of background growth. All transformations were confirmed by colony PCR (primers are listed in Supplementary Table S1). Cells harboring the correct plasmid pairs were cultured overnight (ON) at 28 °C in a liquid synthetic defined (SD) medium lacking leucine and tryptophan (–leu –trp, supplemented only with adenine and histidine), diluted to a density of 2 OD_600_ units/ml, and used to prepare a series of three 10-fold dilutions. From each dilution series, 3 *µ*l-drops were placed on –leu –trp plates (as a control of cell viability and density) and on –leu –trp –his plates (to assay reporter activation), cultured at 28 °C for 12 days (d), and photographed. Each transformed strain was assayed twice.

### Plant morphological observations

For morphological experiments, seeds were stratified 2 d and cultured in the greenhouse. Rosette diameter was measured for 4-week-old (wk-old) plants as the largest diameter across the rosette. For leaf shape, the largest leaf and the 5th or 6th leaf were collected from 7-wk-old rosettes. For shoot number, 9-wk-old plants were used, and each shoot tip (both primary and secondary) was counted. Silique size was judged by taking the 3 largest of the still green siliques from the main shoot of 9-wk-old plants. The same siliques were then opened manually with forceps so that their seed content could be visualized.

Flowers were observed in 9-wk-old plants. Whole inflorescences were removed from the dominant shoot and photographed, then for each inflorescence, all open flowers from youngest to oldest were photographed (flower 1 was the first flower from the top that had fully developed, visible petals). Flowers representing stages 14 to 16 (according to the nomenclature introduced by Smyth et al. 1990) were opened manually with forceps and photographed. Five inflorescences per genotype were observed.

In the case of plate growth assays, the seedlings were cultured on vertical plates, photographed at the indicated time points, and analyzed using the Image J software (v1.53f51). Each experiment was performed twice with similar results.

### Ruthenium red staining

Seeds were imbibed in sterile water for 2 h at room temperature (RT), stained for 1 h in a 0.02% ruthenium red solution, placed on slides in a drop of water and viewed under the microscope. The photos were analyzed using the Image J software (v1.53f51). The experiment was performed twice with similar results.

### In vitro germination of pollen grains

Pollen in vitro germination was conducted as described in Boavida and McCormick (2007), open flowers from 6-wk-old plants were used, the germinating grains were viewed under a microscope after 18 h of incubation at 22 °C. The experiment was performed twice with similar results.

### Aniline blue staining of pollinated pistils

Arabidopsis Col-0 plants were grown for 5.5 wk and flowers were manually emasculated. The pistils were hand-pollinated with pollen from the indicated lines, left for 10 h for germination, fixed ON in Carnoy's solution (60% ethanol, 30% chloroform, and 10% acetic acid), and stained with a procedure slightly modified from that of Lu (2011). The fixative was changed to 70% ethanol, then 50% ethanol, 30% ethanol, and water (10 min at RT in each). Then, the specimens were moved to 1 m NaOH and left covered ON at RT. The pistils were washed with water for 10 min and stained with 0.1% aniline blue in 50 mm KH_2_PO_4_ + K_2_HPO_4_ (pH 7.7) for 3 h in the dark. They were mounted onto microscopic slides in the same phosphate buffer supplied with 50% glycerol and observed under an Eclipse E800 microscope (Nikon Instruments) equipped with a CCD Hamamatsu monochromatic camera. Pollen tube growth (defined as the distance from the pistil stamen to the end of the furthest reaching pollen tube) was measured using the Image J software (v1.53f51) and expressed in relation to the full length of the pistil.

### Starvation sensitivity assays

For carbon starvation assays, the seedlings were grown at standard conditions on solid ½ MS media with 1% sucrose for 7 d and then transferred to plates without sucrose and kept in the dark for another 9 d.

For nitrogen starvation, the seedlings were grown at standard conditions on solid ½ MS media with 0.5% sucrose for 7 d, transferred to plates without nitrogen (½ MS basal salt micronutrient solution (Sigma, M0529) with 3 mm CaCl_2_, 1.5 mm MgSO_4_, 5 mm KCl, 1.25 mm KH_2_PO_4_, 0.5% mannitol, and 3 mm MES, pH 5.6) and kept under the same growth conditions for 14 d. Starvation sensitivity was judged by the extent of leaf chlorosis. The experiment was performed twice with similar results.

### Protein content measurement

Plant material was ground in liquid nitrogen using a mortar and pestle. For assaying 2-wk-old seedlings grown in liquid medium, exactly 100 mg of ground tissue was supplemented with 300 *µ*l of the following buffer: 50 mm Tris-HCl, pH = 8, 150 mm NaCl, 0.1% Igepal CA630, 10% glycerol, 2.5 mm EDTA, 1 mm PMSF and protease inhibitors (Ultra Easy Pack, Roche), and extraction was allowed to proceed for 2 h at 4 °C with shaking. For assaying 4-wk-old rosette leaves, 30 mg of ground tissue were supplemented with 240 *µ*l of 0.1 N NaOH, and extraction was performed for 30 min at RT with agitation. In both cases, cell debris was removed by centrifugation for 10 min at 4,000 g, 4 °C, the supernatant was collected and protein content was measured using the Bradford Assay (BioRad), with the appropriate buffer as control. Each measurement was run in 3 biological replicates.

### Western blotting

Protein extracts for Western blotting were prepared from 100 mg of ground plant material following the same procedure as for protein content measurement. The extracts were mixed with equal amounts of 2 × Laemmlie buffer and heated 5 min at 95 °C. SDS-PAGE was performed on 10% acrylamide gels. Twenty micrograms of protein were loaded per well for anti-NBR1 blots, 15 *µ*g for anti-SKU5 blots, and 25 *µ*g for anti-mCherry. Blotting onto nitrocellulose membranes (Amersham) was conducted using a wet transfer blotting system (BioRad) and controlled by staining with Ponceau S (0.5% in 3% trichloroacetic acid). Membranes were blocked with 3% skimmed milk in TBS + 0.1% Tween-20 (TBST) for 1 h at RT (or ON at 4 °C). The primary antibodies were diluted 1:2000 in TBST with 3% skimmed milk and used for membrane incubation at RT for 1.5 to 2 h: rabbit polyclonal antibody anti-NBR1 was from Agrisera (AS14 2805), rabbit anti-SKU5 primary antibody was a kind gift from Dr. J.C. Sedbrook (Illinois State University, USA), rabbit polyclonal anti-mCherry from Invitrogen (PA5-34974; used at 1:3000). The goat anti-rabbit-IgG-HRP secondary antibody (Sigma, A0545) was used at a concentration of 1:4000, 1 h, RT. TBST was used for membrane washing. Detection was with the SuperSignal West Pico Plus Chemiluminescent Substrate kit (Thermo Scientific) and the exposition of Amersham Hyperfilm MP films.

### Scanning electron microscopy

For SEM observations, seeds or pollen were spilled directly on microscope tables with double-coated carbon tape, then coated with a thin layer of gold with the use of a sputter coater (Polaron SC7620), and examined using a scanning electron microscope LEO 1430VP (Carl Zeiss).

### Transmission electron microscopy

WT and mutant seedlings were cultured in a growth chamber for 7 d on vertical plates with solid ½ MS medium supplemented with vitamins and fixed in 2.5% (w/v) formaldehyde and 2.5% (v/v) glutaraldehyde in a 0.05 m cacodylate buffer pH 7.0 for 4 h at room temperature. Next, the material was rinsed in the same buffer and postfixed in 1% (w/v) osmium tetroxide in a cacodylate buffer at 4 °C overnight, dehydrated in a series of graded acetone and finally embedded in Spurr's low-viscosity resin (Polysciences, Germany). Ultrathin sections (50 to 80 nm) were cut using a diamond knife on a Leica EM UC7 ultramicrotome (Leica, Germany). Then, the sections were stained with Uranyless (Delta Microscopies) and Reynold's lead citrate (Delta Microscopies), and examined on a Tecnai G2 Spirit BioTWIN (FEI, USA) transmission electron microscope at an accelerating voltage of 120 kV. The images have been acquired using a Veleta CCD camera (Olympus Soft Imaging Solutions) and Radius software. TEM analyses were done at the Bioimaging Laboratory of the Faculty of Biology, University of Gdańsk, Poland.

### Confocal microscopy

WT and mutant seedlings carrying the [P_UBI_::mCherry-ATG8E] transgene were cultured in a growth chamber for 5 d on horizontal plates with solid ½ MS medium supplemented with vitamins and 1% sucrose. The seedlings were then transferred to 6-well plates, where 10 seedlings each were incubated 2.5 to 3 h in the dark at RT with agitation in 3 ml of liquid medium (same as above) supplemented with either 3 *µ*l of 1 mm Concanamycin A (Sigma, C9705) dissolved in DMSO or with 3 *µ*l DMSO alone (control). The seedlings were then mounted on microscopy slides and root cells from the elongation zone were viewed. Imaging of the mCherry fluorophore was carried out on an inverted microscope (Nikon, TE-2000E) with a confocal laser-scanning mode EZ-C1. mCherry fluorescence was excited with light emitted by a Green He-Ne Laser set at 30% (1.0 mW, 543 nm; Melles Griot, USA) then collected with a 605/75 nm single band pass emission filter (BrightLine, Semrock) and displayed in false red. Cells have been imaged using a 60 × oil immersion objective (Nikon, CFI Plan Apochromat NA 1.4). All confocal adjustments (the pinhole diameter set to 30 *µ*m, the gain of the detector set to 8.4, the time dwell set to 10.08 µs) were the same during the experiment. Confocal sections were collected at 0.5 *µ*m intervals, resulting in stacks encompassing the fixed volume of: 120 *µ*m × 75 *µ*m × 20 *µ*m. Puncta of diameter 0.4 to 2.5 *µ*m were counted manually from the obtained image stacks. For each genotype/condition, 8 to 16 image stacks taken from 4 to 9 individual seedlings were scored. The full experiment was repeated twice with similar results.

### Reverse transcription-quantitative PCR (RT-qPCR)

One hundred milligrams of plant material ground in liquid nitrogen was used for RNA isolation with the GeneJET Plant RNA Purification Mini Kit (Thermo Scientific). The samples were digested with the RapidOut DNA Removal Kit (Thermo Scientific), and 0.5 *μ*g of RNA was reverse-transcribed using the RevertAid First Strand cDNA Synthesis Kit (Thermo Scientific). The obtained cDNA was quantified by qPCR using SG qPCR Master Mix (2×) plus ROX Solution (EURx, Gdansk, Poland) and a StepOnePlus Real-Time PCR System (Applied Biosystems). The cDNA was diluted 10-fold and 10 *μ*l was used in a total reaction volume of 25 *μ*l per well. All primers used for RT-qPCR analyses are listed in Supplementary Table S1. The gene encoding PROTEIN PHOSPHATASE 2A SUBUNIT A3 (PP2A) was used as internal reference. The expression of each gene was examined in 2 technical and 3 biological replicates. The relative expression levels were determined using the 2^−ΔΔCt^ method and normalized to expression in WT plants.

### Isolation of polyisoprenoids, sterols, and fatty acids from Arabidopsis tissue

Plant material (batch of seedlings or collected from several plants; for polyisoprenoids ca. 1 g of fresh mass, except the experiment with lyophilized seedlings where 200 to 400 mg of dry material were used, for sterols 0.5 g of fresh mass) was either ground in liquid nitrogen and transferred to 20 ml of a 1:1 (v/v) chloroform/methanol mixture (C/M), or homogenized directly in 20 ml of the same mixture with an Ultra-Turrax apparatus (IKA Labortechnik). The samples were supplemented with the appropriate internal standard (10 *µ*g of prenol-14 for polyisoprenoid isolation, 5 *µ*g of cholestan-3β-ol for sterol isolation) and agitated for 48 h at room temperature. The C/M extracts were filtered and evaporated under a stream of nitrogen. The obtained lipids were resuspended in 5 ml of hydrolysis mixture (7.5% (w/v) KOH in ethanol: toluene: water, 4:5:1 by volume) and incubated for 1 h at 96 °C. After cooling, 5 ml of each water and hexane were added, and the mixture was vortexed. After phase separation, the organic phase was collected, and extraction of the aqueous phase was repeated twice with 5 ml hexane each. The pooled organic phases were evaporated under a stream of nitrogen and dissolved in 500 *µ*l of hexane. The extracts were loaded onto 2 ml-silica gel columns in hexane.

For polyisoprenoid isolation, the columns were eluted with 10 ml 2% diethyl ether in hexane and 20 ml of 15% diethyl ether in hexane. The latter fraction was evaporated and dissolved in 100 *µ*l of 2-propanol; all of the sample was used for HPLC/UV analysis.

For sterol isolation, the columns were eluted with 10 ml each of 2% and 10% diethyl ether in hexane, and next with 10 ml each of 15% and 30% diethyl ether in hexane. The 2 final fractions were evaporated and dissolved in 600 *µ*l of a mixture of diethyl ether/methanol (1/1, v/v); both fractions were analyzed separately; 3 *µ*l of each sample were used for GC/MS analysis.

For fatty acid isolation, seedlings were ground in liquid nitrogen and 1 g of material was transferred to 4 ml of a 2:1 (v/v) C/M, agitated 1 h at room temperature, the chloroform phase was collected, material was reextracted overnight with the same amount of C/M and again collected. The C/M extracts were rinsed with 2 ml of 5 m NaCl, filtered, and evaporated under a stream of nitrogen. The obtained lipids were suspended in 600 *µ*l of C/M.

Three independent biological replicates were run for each experiment.

### HPLC/UV analysis of polyisoprenoid alcohols

Polyisoprenoid alcohols were analyzed by HPLC/UV as described previously (Jozwiak et al. 2013), with modifications. Runs were performed on a 4.6 × 75 mm ZORBAX XDB-C18 (3.5 *μ*m) reversed-phase column (Agilent) using a Waters dual-pump apparatus, a Waters gradient programmer, and a Waters Photodiode Array Detector (spectrum range: 210 to 400 nm). The chain length and identity of lipids were confirmed by comparison with external standards of a polyprenol mixture (Prenol-9–25) and dolichol mixture (Dolichol-17–21). Quantitative determination of polyisoprenoids was performed using the internal standards. Integration of the HPLC/UV chromatograms was performed with the Empower software (Waters). All polyprenol and Dol standards were from the Collection of Polyprenols, Institute of Biochemistry and Biophysics, Polish Academy of Sciences (Warsaw, Poland).

### GC/MS analysis of sterols

The sterol-containing fractions were analyzed using GC/MS (7890 A Agilent gas chromatograph with an FID, equipped with a 5975 C VL MSD—Perlan, Warszawa). Samples dissolved in a mixture of diethyl ether/methanol (1/1, v/v) were applied in a volume of 3 *µ*l using a 1:3 split injection on the Ultra-Inert GC column HP-5MS UI (30 m × 0.25 mm i.d.; film thickness 0.25 *µ*m—Agilent Technologies, Santa Clara, CA, USA). The carrier gas was helium at a flow rate of 1 ml/min. The following program was carried out: 160 °C for 2 min, then an increase of 5° per minute up to 280 °C, which were held for 24 min. The following settings were used: inlet and FID temperature: 290 °C, MS transfer line temperature: 275 °C, quadrupole temperature: 150 °C, ion source temperature: 230 °C, EI: 70 eV, m/z range: 33 to 500, FID gases: H_2_ from a hydrogen generator at 30 mL/min and air at 400 mL/min. Compound identification was based on comparisons of the obtained mass spectra with library data (Wiley 9th Ed. and NIST 2008 Lib SW Version 2010) and on comparisons of the obtained retention times and mass spectra with those for external standards. FID chromatograms were integrated using the ChemStation Integrator software; sterol content was normalized using the applied internal standard (cholestan-3β-ol).

### GC/FID analysis of fatty acids

Total lipid extracts (0.2 ml from original samples) were dried and the corresponding fatty acid methyl esters (FAMEs) were prepared according to the protocol described by Patel et al. (2016, 2018), with modifications. Briefly, lipid samples were transmethylated by adding NaOH (v/v, 1% in methanol; 1 ml), followed by heating at 55 °C for 15 min. Thereafter, 2 ml methanolic HCl (v/v, 5%) was added, and the samples were further heated at 55 °C for 15 min. Samples were cooled on ice, water (1 ml) was added, and FAMEs were extracted with hexane (2 ml) by vigorous shaking. Samples were allowed to stand for 10 to 15 min, and then the upper hexane layer was removed and concentrated under nitrogen. Pentanoic acid (Sigma-Aldrich, St. Louis, MO, USA), was added (10 *µ*l, 6.25 mg/ml solution in CHCl_3_:MeOH 2:1, v/v) as an internal standard. The FAMEs were analyzed on a Varian GC 450 gas chromatograph equipped with a Select FAME capillary column (50 m × 0.25 mm × 0.25 *μ*m; Varian Select Column) coupled to a flame ionization detector (FID). The injector and detector temperatures were kept at 250 °C. The initial column temperature was 80 °C and was maintained for 0.5 min. The temperature was then increased to 220 °C at a rate of 20 °C min^-1^ and maintained for 7 min. The gas flow rates used were 1.0 ml/min carrier gas (helium), 29 ml/min make-up gas (N_2_), and 300 ml/min flame gas (H_2_). The sample split mode was 1:100 and the volume of injection was 1 *μ*l. Certified reference materials: FAME MIX C8 to C24 (Supelco, USA, purchased from Sigma-Aldrich, St. Louis, MO, USA), methyl 7(Z),10(Z),13(Z)-hexadecatrienoate, methyl and 7(Z),10(Z)-hexadecadienoate (Larodan, Sweden), were used to identify FAMEs. The peak areas were determined with the software Workstation version 5.0 (Varian).

### Structural modeling of the TRAPPIII complex

Structural modeling of the *A. thaliana* TRAPPIII complex was done using the newest version of Alphafold (Abramson et al. 2024) (ver.3, accessed online). The obtained model was visually inspected, especially in comparison to known 3D structures of TRAPP: TRAPPIII partial complex from *D. melanogaster* (pdb|7b6r, Galindo et al. 2021) and TRAPPII from *S. cerevisiae* (pdb|7ea3, Mi et al. 2022). Sequences of animal and plant homologs of the TRAPPC8 and −C11 proteins were obtained using blastp searches (Altschul et al. 1997) run against a locally downloaded nr (nonredundant) protein sequence database. The sequences were aligned using the MAFFT program (Katoh and Standley 2014) set-up for the “auto” option to choose the optimal alignment strategy.

### Statistical analysis

Statistical analysis was performed with GraphPad Prism 9.5.1 (GraphPad, San Diego, CA, USA) software. For inheritance analysis, the χ2 test or the binomial test was performed against Mendelian H_0_ hypotheses, as described in Table 1. For analysis of root length, seed axis length, or pollen tube length, the structures were measured using ImageJ and compared pairwise using unpaired *t*-tests with Welch's correction against the H_0_ hypotheses that the lengths are equal. Protein and isoprenoid content, as well as RT-qPCR results, were compared pairwise using unpaired *t*-tests against the H_0_ hypotheses that the content is unchanged. The numbers of mCherry puncta in the autophagy experiment were analyzed with unpaired *t*-tests with Welch's correction. The box-and-whiskers plots used in this paper show min to max values with median indicated and with whiskers representing the 25th and 75th percentile, the bar graphs display mean values with standard deviations. Results with *P*-values above 0.05 were classified as statistically insignificant.

### Accession numbers

Sequence data used in this article can be found in The Arabidopsis Information Resource database (http://www.arabidopsis.org) under accession numbers: TRAPPC8 (AT5G16280), TRAPPC11 (AT5G65950), TRAPPC12 (AT4G39820), TRAPPC13 (AT2G47960), TRAPPC2 (AT1G80500), TRAPPC2(L) (AT2G20930), ATG8E (AT2G45170), CNX1 (AT5G61790), BiP2 (AT5G42020), BiP3 (AT1G09080), ERDJ3A (AT3G08970), PDI6 (AT1G77510), bZIP60 (AT1G42990), NBR1 (AT4G24690), HMGR1 (AT1G76490), HMGR2 (AT2G17370), MVK (AT5G27450), DXS (AT4G15560), DXR (AT5G62790), MCT (AT2G02500), FPS1 (AT5G47770), FPS2 (AT4G17190), CPT3 (AT2G17570), PPRD2 (AT2G16530), EVN (AT3G45040), and PP2AA3 (AT1G13320).

## Supplementary Material

kiaf042_Supplementary_Data

## Data Availability

The structural model of the *Arabidopsis thaliana* TRAPPIII complex is available in ModelArchive at https://www.modelarchive.org/doi/10.5452/ma-rt1m0. Imaging data is available in BioImage Archive at https://doi.org/10.6019/S-BIAD1075 (confocal microscopy data) and https://doi.org/10.6019/S-BIAD1444 (TEM data). All other data underlying this study is available in BioStudies: https://doi.org/10.6019/S-BSST1721.

## Supplementary data

The following materials are available in the online version of this article.

**Supplementary Figure S1.** Binary interactions between the TRAPPC8 and −C11 subunits assayed using the yeast Y2H system.

**Supplementary Figure S2.** Domain analysis and structural modeling of *A. thaliana* TRAPPIII.

**Supplementary Figure S3.** Analysis of the insertion lines SALK_124093 (*trappc8-1*) and SALK_130580 (*trappc8-2*).

**Supplementary Figure S4.** The mutations *trappc8-1* and *trappc8-2* are allelic.

**Supplementary Figure S5.** Phenotypic analysis of the mutant lines *trappc11-6* and *trappc11-7*.

**Supplementary Figure S6.** Quantitative data for starvation assays and protein content measurements of *trappc8-1* and *trappc8-2* mutants.

**Supplementary Figure S7.** Expression of the gene *NBR1* and the corresponding protein in *trappc8-1* and *trappc8-2* plants.

**Supplementary Figure S8.** Western blot confirming expression of the mCherry-ATG8E hybrid protein.

**Supplementary Figure S9.** Growth of seedlings at various concentrations of ER-stress-inducing agents.

**Supplementary Figure S10.** Western blotting of SKU5 protein.

**Supplementary Figure S11.** Dol levels in *trappc8-1* plants.

**Supplementary Figure S12.** RT-qPCR analysis of the expression of genes from the dolichyl phosphate biosynthesis pathway in the mutants *trappc8-1* and *trappc8-2*.

**Supplementary Figure S13.** Dol levels and TUN sensitivity of autophagic mutants.

**Supplementary Figure S14.** Dol levels in *trappc11* mutant plants.

**Supplementary Table S1.** Primers used in this study.

## Dive Curated Terms

The following phenotypic, genotypic, and functional terms are of significance to the work described in this paper:

pin1 Gramene: AT1G73590
pin1 Araport: AT1G73590

SKU5 Gramene: AT4G12420
SKU5 Araport: AT4G12420

ATG2 Gramene: AT3G51800
ATG2 Araport: AT3G51800

ARF2 Gramene: AT5G62000
ARF2 Araport: AT5G62000

TGN Gramene: trans-Golgi network
TGN Araport: trans-Golgi network

ATG5 Gramene: AT5G17290
ATG5 Araport: AT5G17290

ruthenium red CHEBI: CHEBI:34956

ER Gramene: endoplasmic reticulum
ER Araport: endoplasmic reticulum
