# Supplementary material for: The TRAPPC8/TRS85 subunit of the Arabidopsis TRAPPIII tethering complex regulates endoplasmic reticulum function and autophagy
Source: Plant Physiol. 2025 Mar 14;197(3):kiaf042. doi: 10.1093/plphys/kiaf042 (PMC11907232; doi:10.1093/plphys/kiaf042)
Supplement: kiaf042_Supplementary_Data [file kiaf042_supplementary_data.zip › SupplementaryDataMHS.pdf]

Supplementary Data for

"The TRAPPC8/TRS85 subunit of the Arabidopsis TRAPPIII tethering complex regulates endoplasmic reticulum function and autophagy" (2024)

by Marta Hoffman-Sommer, Natalia Piłka, Anna Anielska-Mazur, Julita Nowakowska, Małgorzata Kozieradzka-Kiszkurno, Cezary Pączkowski, Małgorzata Jemioła-Rzemińska, Kamil Steczkiewicz, Yasin Dagdas, Ewa Swiezewska

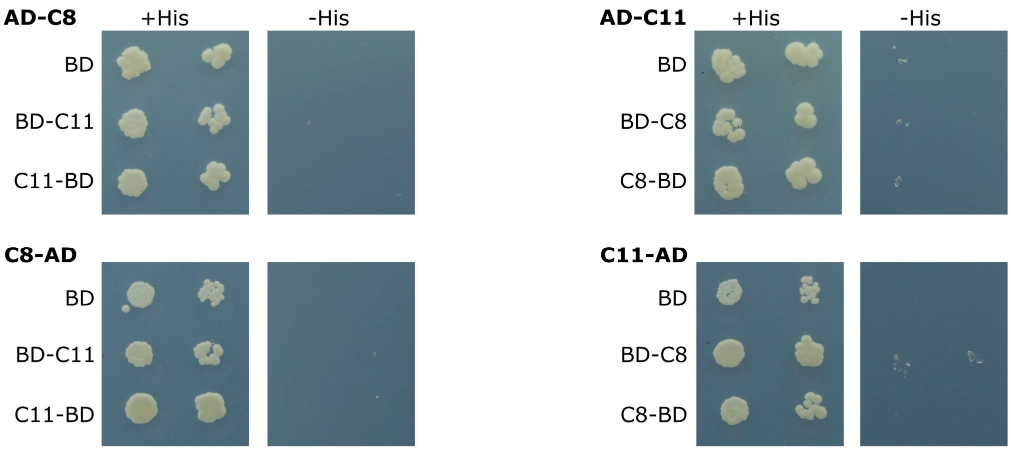

Supplementary Fig. S1. Binary interactions between the TRAPPC8 and -C11 subunits assayed using the yeast two-hybrid system. For each panel, one hybrid protein is indicated above the panel and the other to the left. Abbreviations used: AD – Gal4 activation domain, BD – Gal4 DNA-binding domain. The names of TRAPP subunits are shortened to subunit numbers. +His marks control plates, -His are test plates showing reporter activation. In this assay the subunits TRAPPC8 and TRAPPC11 did not interact in any combination.

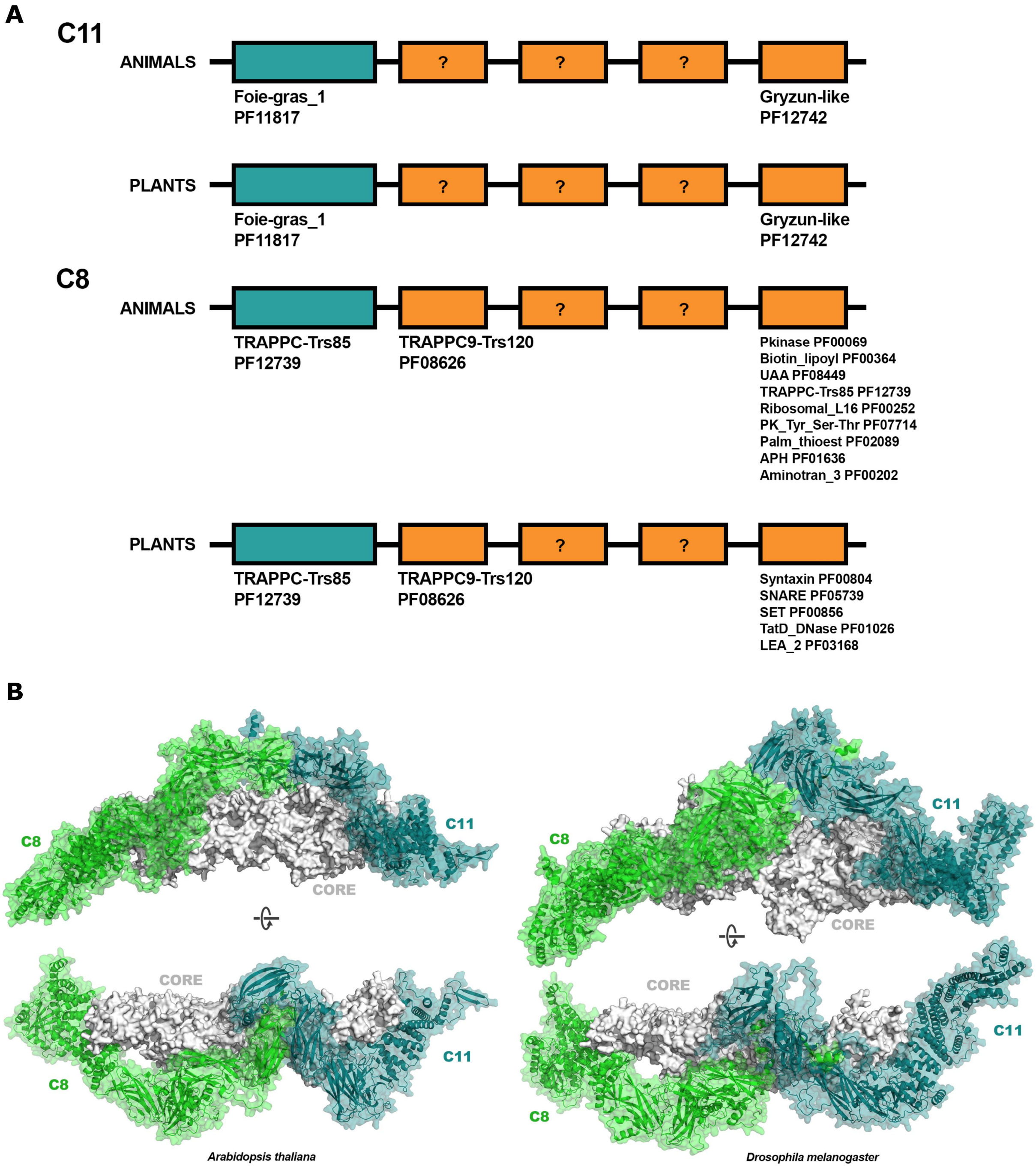

Supplementary Fig. S2. Domain analysis and structural modeling of *A. thaliana* TRAPPIII. (A) Domain architecture of plant and animal TRAPPC8 and -C11. The N-terminal domains are highly conserved and map well onto known *pfam* domains (blue); all other domains display structural similarity to each other (orange). For C11 also the C-terminal Gryzun-like domain is well-defined. For C8 the second domain is well-defined and is present also in the TRAPPII complex subunit TRAPPC9/TRS120, while the C-terminal domain shows more variability and maps weakly onto

many known domains. Other detected domains are of unknown function, but all clearly retain the Ig-like structural fold. (B) Comparison of structural models for *A. thaliana* and *D. melanogaster* TRAPPIII, omitting the C12-C13 dimer for clarity. The TRAPP core is depicted in white, the C8 subunits in green, and C11 subunits in blue.

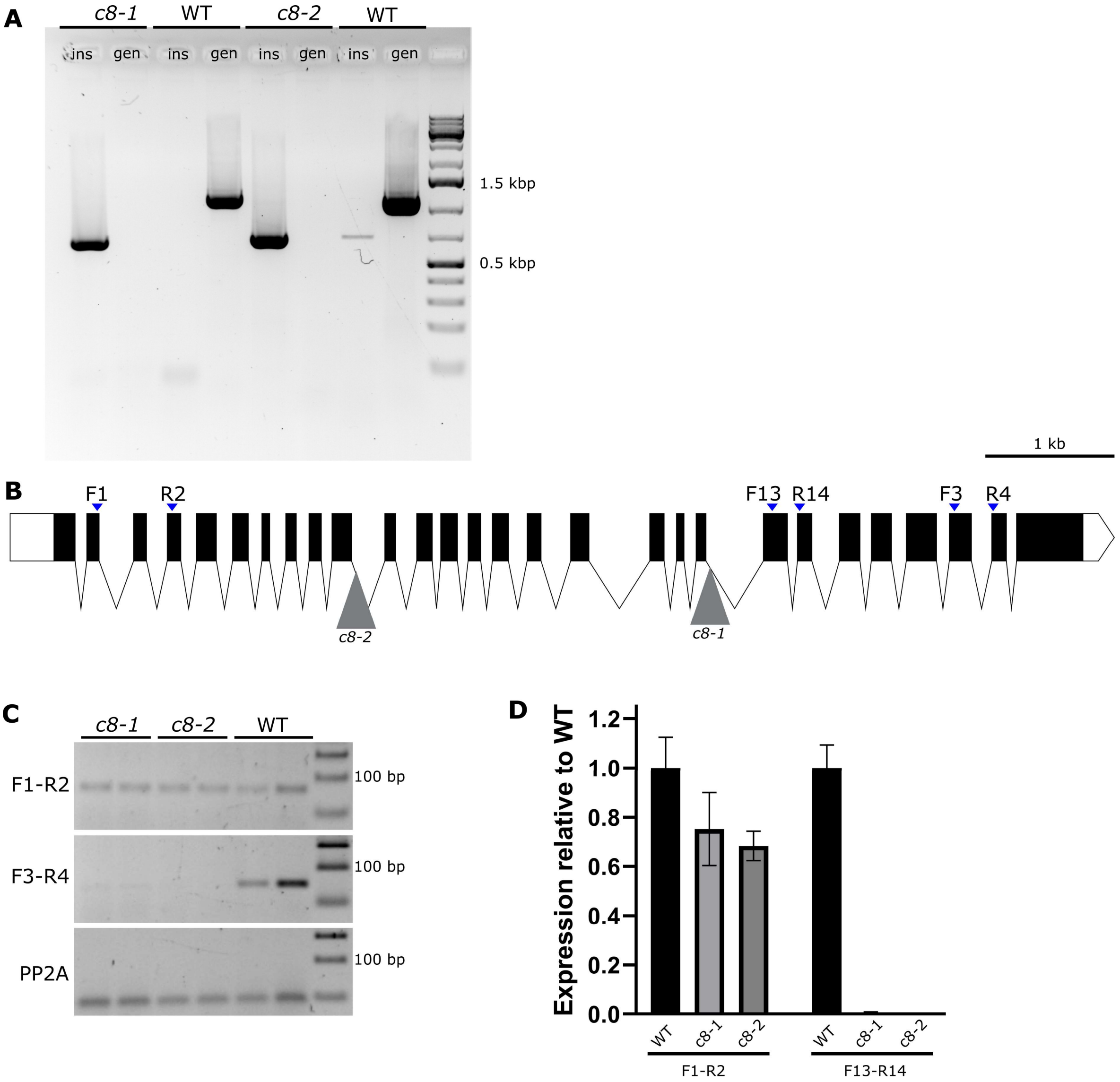

Supplementary Fig. S3. Analysis of the insertion lines SALK_124093 (*trappc8-1*) and SALK_130580 (*trappc8-2*). WT – wild-type, *c8-1* – *trappc8-1*, *c8-2* – *trappc8-2*. (A) Genotyping of the isolated homozygotic mutant plants. ins – confirmation of allele with T-DNA insert, gen – confirmation of genomic allele. (B) Diagram of the intron-exon structure of the *AtTRAPPC8* gene. Each box represents an exon. The positioning of insertions in the mutant lines (gray triangles below the diagram) and of primer pairs used for semi-quantitative PCR and RT-qPCR (F1-R2, F13-R14, F3-R4; blue triangles above the diagram) is shown. White boxes indicate untranslated regions (UTR). (C) Results of semi-quantitative PCR analyzing the expression of the mutated *TRAPPC8* genes. *PP2A* served as an expression control. (D) RT-qPCR of the analyzed lines. The primer pairs used are indicated below the graph. Three biological replicates were performed and mean values and standard deviations are shown. RNA for experiments shown in C and D was extracted from rosette leaves of 4-week old plants grown in soil.

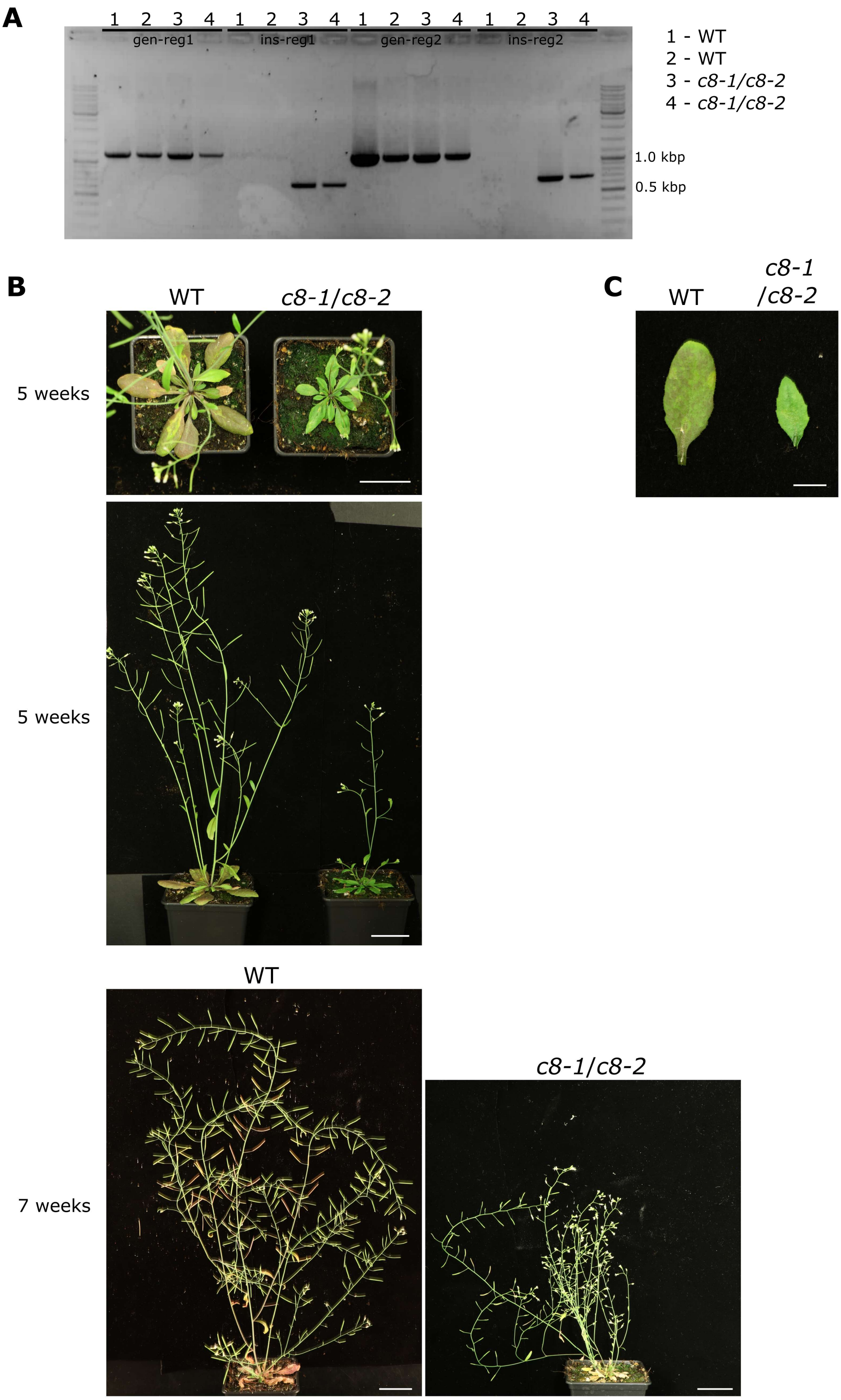
A
1 2 3 4 1 2 3 4 1 2 3 4 1 2 3 4
gen-reg1
ins-reg1
gen-reg2
ins-reg2
1 - WT
2 - WT
3 - c8-1/c8-2
4 - c8-1/c8-2
1.0 kbp
0.5 kbp
B
WT
c8-1/c8-2
5 weeks
5 weeks
WT
7 weeks
c8-1/c8-2
C
WT
c8-1
/c8-2

Supplementary Fig. S4. The mutations *trappc8-1* and *trappc8-2* are allelic. (A) Genotyping of four plants from the progeny of *trappc8-1*$^{+/-}$ × *trappc8-2*$^{+/-}$. **gen-reg1** – PCR with primers for the genomic version in region 1 of the *TRAPPC8* gene (corresponding to where the *c8-1* mutation is located); **ins-reg1** – PCR with primers detecting the insert in region 1; **gen-reg2** – PCR with primers for the genomic version in region 2 of the gene (corresponding to where the *c8-2* mutation is located); **ins-reg2** – PCR with primers detecting the insert in region 2. The plants 1 and 2 display a wild-type (WT) genotype, 3 and 4 are double mutants *trappc8-1*/*trappc8-2* (*c8-1*/*c8-2*). (B) Overall morphology of WT and double mutant plants cultured in soil for 5 and 7 weeks. Scale bars: 3 cm. (C) Representative rosette leaves from 5-week old plants. Scale bar: 1 cm.

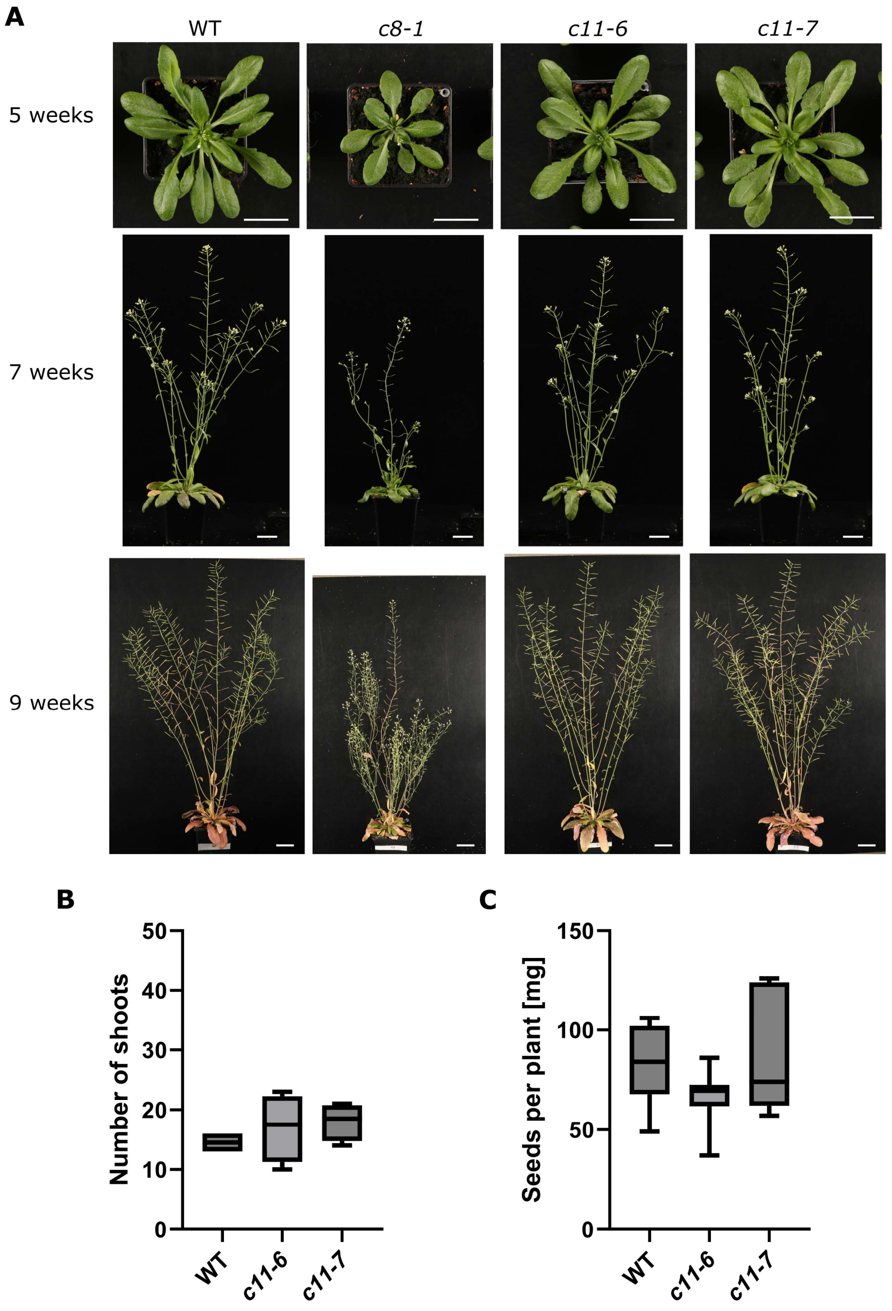

Supplementary Fig. S5. Phenotypic analysis of the mutant lines *trappc11-6* and *trappc11-7*. (A) Overall morphology of WT and mutant plants cultured in soil. The mutant *trappc8-1* is shown for comparison. Scale bars: 3 cm. (B) Quantification of total number of shoots (primary and secondary) of 9-week old plants. 4 plants were scored for each genotype. (C) Quantification of amount of seeds produced per plant. The seeds of 7-8 individual plants were weighed for each genotype. In B and C, the box-and-whiskers plots show min to max values with median indicated and whiskers representing the 25th and 75th percentile. Using pairwise (mutant vs WT) unpaired t-tests with Welch's correction, the differences between WT and mutants were statistically insignificant in B and C.

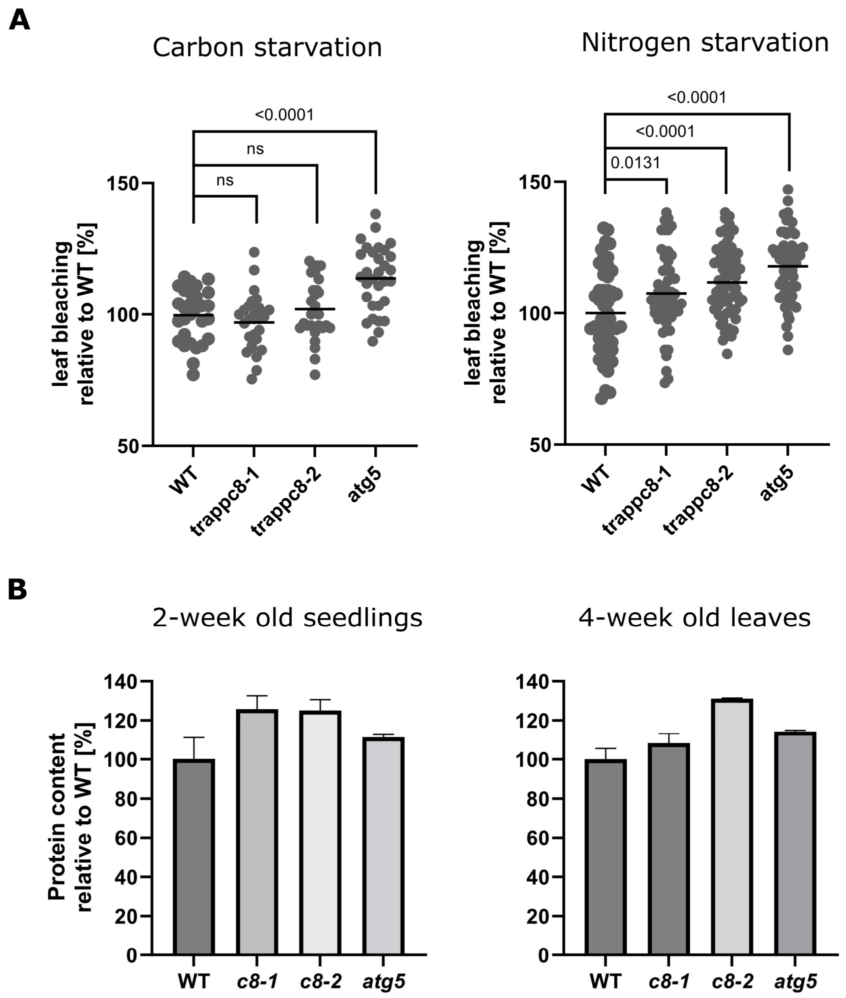

Supplementary Fig. S6. Quantitative data for starvation assays and protein content measurements of *trappc8-1* and *trappc8-2* mutants. (A) Leaf bleaching of seedlings from the starvation experiment presented in Figure 8A was analyzed semi-quantitatively using ImageJ, by converting the images to grayscale and extracting the mean intensity from a leaf fragment of defined size. The data was normalized to the average intensity of leaves from the WT control. For carbon starvation, 28-30 seedlings were scored per genotype, for nitrogen starvation 53-62 seedlings. The horizontal bars indicate the median for each result set. P values for unpaired t-tests with Welch's correction are depicted. ns – non-significant (P value > 0.05). (B) Protein content of *trappc8-1* and *trappc8-2* plants. Protein content per mg of tissue was assayed in 2-week old seedlings grown in liquid culture and in rosette leaves of 4-week old, soil-grown plants of the indicated genotypes. Three biological replicates were performed, mean values and standard deviations are shown. Unpaired t-tests with Welch's correction showed no statistically significant differences between WT and mutant plants, also for the control line *atg5*.

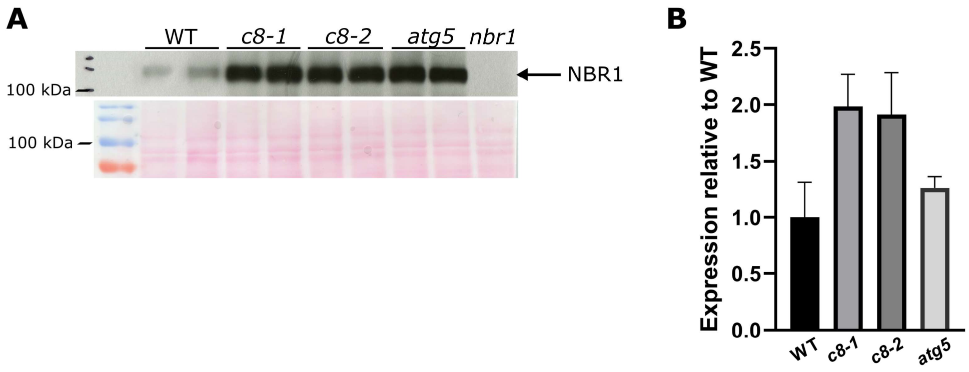

Supplementary Fig. S7. Expression of the gene *NBR1* and the corresponding protein in *trappc8-1* and *trappc8-2* plants. (A) Plate-grown 2-week old seedlings of the indicated genotypes were subjected to protein extraction and Western blot analysis with an anti-NBR1 antibody. Ponceau S staining of the membrane is shown below to demonstrate gel loading. (B) Liquid-grown 2-week old seedlings of the indicated genotypes were subjected to RNA extraction and RT-qPCR analysis. Three biological replicates were performed, mean values and standard deviations are shown. The levels of transcripts for the *NBR1* gene are shown in relation to the level for WT seedlings.

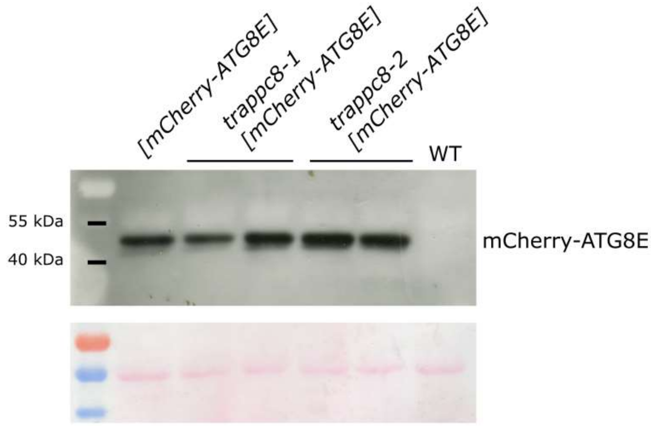

Supplementary Fig. S8. Western blot confirming expression of the mCherry-ATG8E hybrid protein. Protein extracts were prepared from leaves of 5-week old plants grown in soil. The membrane was probed with an anti-mCherry antibody. The detected bands correspond to the expected molecular mass of mCherry-ATG8E, which is 46 kDa. Ponceau S staining of the membrane is shown below to demonstrate gel loading.

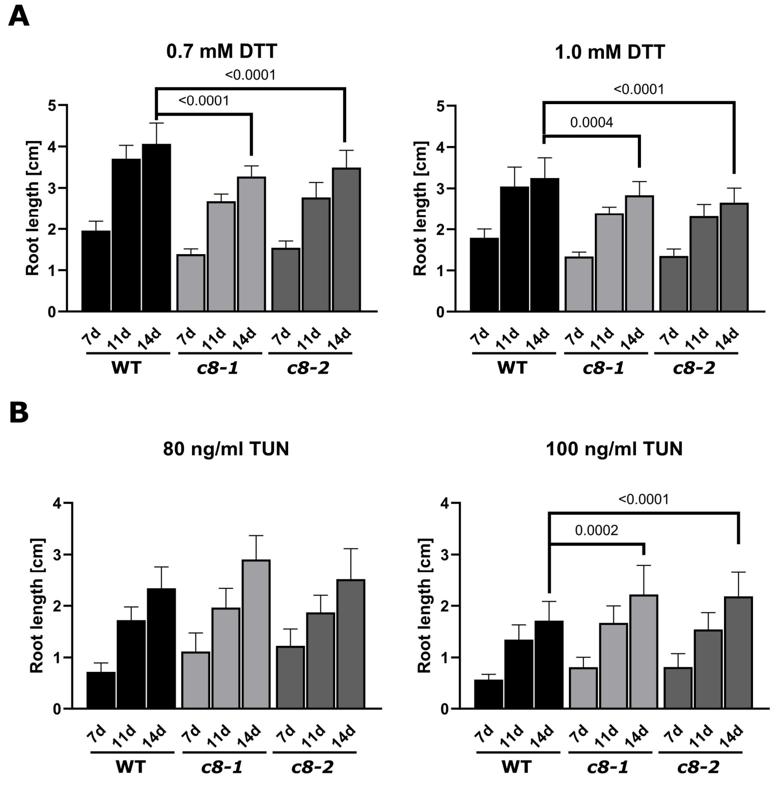

Supplementary Fig. S9. Growth of seedlings at various concentrations of ER-stress inducing agents. WT and *trappc8* seeds were sowed on plates containing the indicated concentrations of (A) dithiothreitol (DTT) or (B) tunicamycin (TUN) in the growth medium. Seedlings (22-32 seedlings per genotype for the DTT experiment, 28-30 for the TUN experiment) were photographed after 7, 11 and 14 days of growth and root length was measured using ImageJ. Mean values with standard deviations are shown. P values for unpaired t-tests with Welch's correction are depicted on the graphs. WT – wild-type, *c8-1* – *trappc8-1*, *c8-2* – *trappc8-2.*

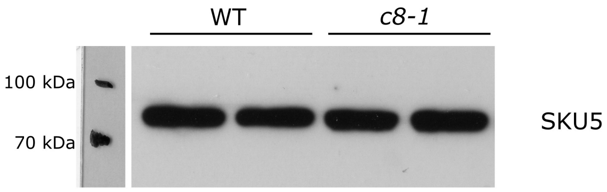

Supplementary Fig. S10. Western blotting of SKU5 protein. Protein extracts were prepared from 2-week old seedlings grown in liquid culture. Anti-SKU5 primary antibodies were used.

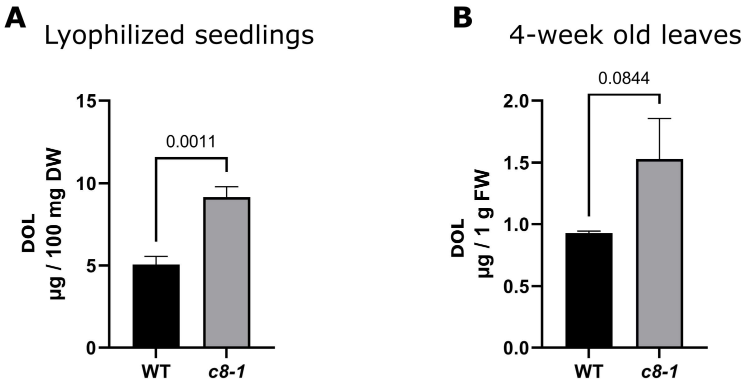

Supplementary Fig. S11. Dolichol levels in *trappc8-1* plants. Accumulation of dolichols (-15, -16 and -17) was assayed by HPLC/UV analysis of isoprenoids extracted from (A) lyophilized seedlings and (B) 4-week old leaves of WT plants and *trappc8-1* mutants. DW – dry weight, FW – fresh weight. Data is from three biological replicates. Mean values with standard deviations are shown. Results were compared pairwise (mutant versus corresponding WT) using unpaired t-tests with Welch's correction. P values are depicted on the graphs. WT – wild-type, *c8-1* – *trappc8-1*.

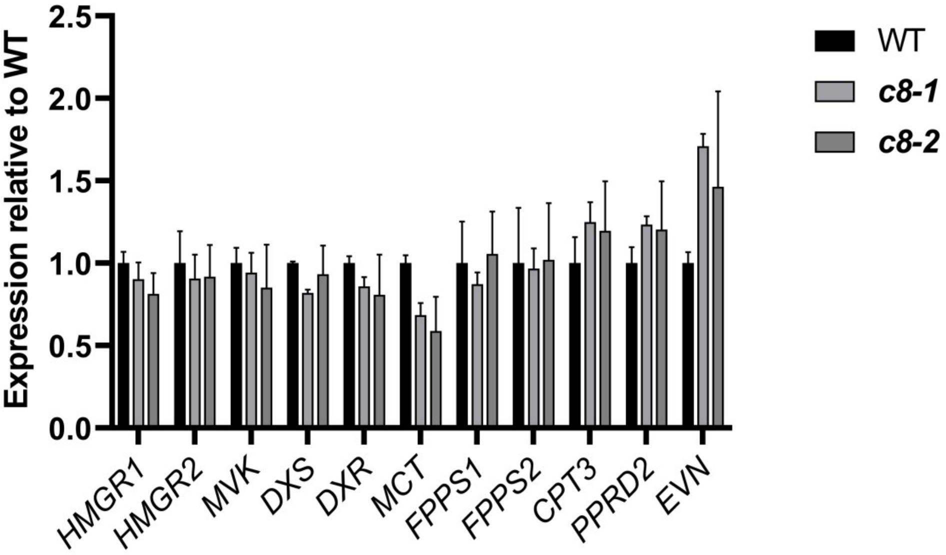

Supplementary Fig. S12. RT-qPCR analysis of the expression of genes from the dolichyl phosphate biosynthesis pathway in the mutants *trappc8-1* and *trappc8-2*. RNA for this experiment was prepared from 2-week old seedlings grown in liquid culture. Three biological replicates were analyzed for each genotype. Bars represent means with standard deviations. Assayed genes encode the following enzymes: *HMGR1* – 3-hydroxy-3-methylglutaryl-CoA reductase 1 (MVA pathway); *HMGR2* – 3-hydroxy-3-methylglutaryl-CoA reductase 2 (MVA pathway); *MVK* – mevalonate kinase (MVA pathway); *DXS* – 1-deoxy-d-xylulose-5-phosphate synthase (MEP pathway); *DXR* – 1-deoxy-d-xylulose-5-phosphate reductoisomerase (MEP pathway); *MCT* – 4-diphosphocytidyl-2C-methyl-D-erythritol-4-phosphate synthase (MEP pathway); *FPPS1* – farnesyl diphosphate synthase 1; *FPPS2* – farnesyl diphosphate synthase 2; *CPT3* – *cis*-prenyltransferase 3; *PPRD2* – polyprenol reductase 2; *EVN* – dolichol kinase. WT – wild-type, *c8-1 – trappc8-1*, *c8-2 – trappc8-2*.

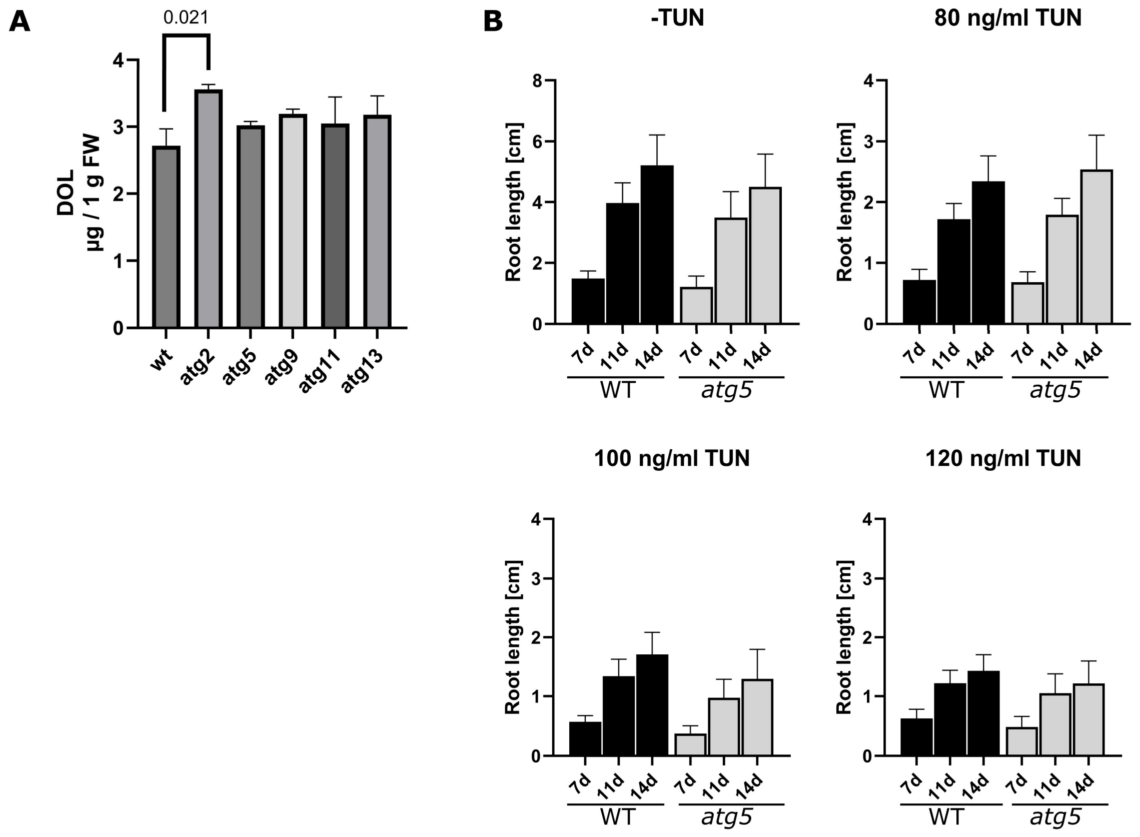

Supplementary Fig. S13. Dolichol levels and tunicamycin sensitivity of autophagic mutants. (A) Dolichol levels were assayed by HPLC/UV analysis of isoprenoids extracted from 2-week old liquid-grown seedlings of the indicated genotypes. Data is from three biological replicates. Mean values with standard deviations are shown. Results were compared pairwise (each mutant versus corresponding WT) using unpaired t-tests with Welch's correction against the H_0 hypotheses that the content is unchanged. The only significant P value is depicted on the graph. (B) WT and *atg5* seeds were sowed on plates containing the indicated concentrations of tunicamycin (TUN) in the growth medium. Seedlings were photographed after 7, 11 and 14 days of growth and root length was measured using ImageJ. 30 seedlings were scored for WT, 12 for the *atg5* line. Mean values with standard deviations are shown. Results were compared pairwise (each mutant versus corresponding WT) using unpaired t-tests with Welch's correction. No significant differences between WT and mutant seedlings were observed.

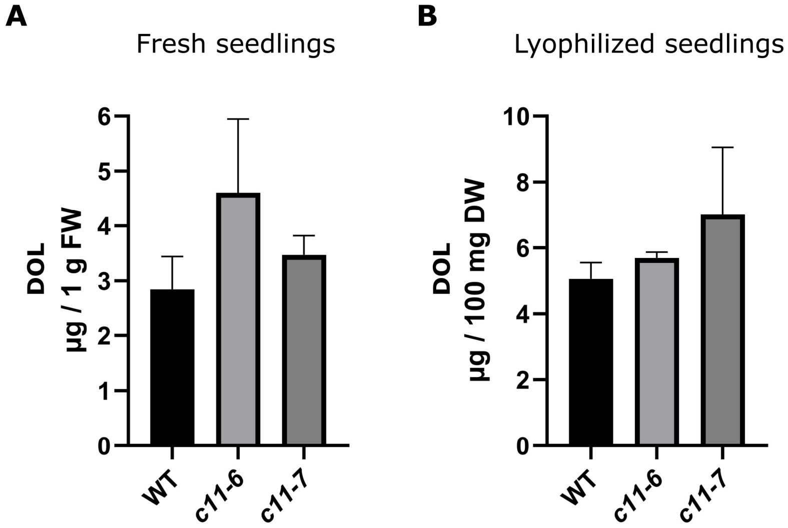

Supplementary Fig. S14. Dolichol levels in *trappc11* mutant plants. Accumulation of dolichols (sum of dolichol-15, -16 and -17) was assayed by HPLC/UV analysis of polyisoprenoids extracted from seedlings of WT and *trappc11* lines cultured for 2 weeks in liquid medium and (A) used freshly or (B) lyophilized. FW – fresh weight, DW – dry weight. Data is from three biological replicates. Mean values with standard deviations are shown. Results were compared pairwise (each mutant versus corresponding WT) using unpaired t-tests with Welch's correction. In this experiment all differences between lines were statistically not significant. WT – wild-type, *c11-6* – *trappc11-6*, *c11-7* – *trappc11-7*.

Supplementary Table S1. Primers used in this study.

Primer name	Sequence (5’→3’)	Purpose
S124093L	TGATCAATTGAACCCTCAAGC	Genotyping of SALK_124093
S124093R	TTTCAGAGTCGGTGAAATTGG	Genotyping of SALK_124093
S130580L	ACTTGGATGTGCTCTCACTGG	Genotyping of SALK_130580
S130580R	ATGGTTCACCGCATCACTTAC	Genotyping of SALK_130580
W01205BL	ACCACCACCCACATCAACTAG	Genotyping of WiscDSLoxHS012_05B
W01205BR	TTTTCACTTGAAATGGTTGGC	Genotyping of WiscDSLoxHS012_05B
I118f07L	GCAACCAATTCGAATTCTTTG	Genotyping of SAIL_118F07
I118f07R	CTGGAATTGAAGAAGAGCACG	Genotyping of SAIL_118F07
atg5-LP	ATTTGCTATTTGTTTGGCACG	Genotyping of SAIL_129_B07
atg5-RP	TACCGTTCATGACAGAGGTCC	Genotyping of SAIL_129_B07
SAIL-LB1	TAGCATCTGAATTTCATAACCAATCT CGATACAC	Genotyping of SAIL lines
LBb1.3	ATTTTGCCGATTTCGGAAC	Genotyping of SALK lines
WDLH-L4	TGATCCATGTAGATTTCCCGGACATG AAG	Genotyping of WiscDSLoxHS lines
mCH-8E	ACGAGTTCGAGATCGAGG	Genotyping of lines with transgene *[mCherry-ATG8E]*
ATG8E-R	ATTGAAGAAGCACCGAATGT	Genotyping of lines with transgene *[mCherry-ATG8E]*
C8-BP-F	GGGGACAAGTTTGTACAAAAAAGCA GGCTTC**ATG**GTGGAGCCGGTGAACTC TT	Cloning of AtTRAPPC8 into the pDONR201 vector
C8-BP-R	GGGGACCACTTTGTACAAGAAAGCTG GGTC**CTC**AGACTGAAGAACAGTAAG G	Cloning of AtTRAPPC8 into the pDONR201 vector
C2-BP-F	GGGGACAAGTTTGTACAAAAAAGCA GGCTTC**ATG**ATCGTTTGCGTCGCCGT C	Cloning of AtTRAPPC2L into the pDONR201 vector
C2-BP-R	GGGGACCACTTTGTACAAGAAAGCTG	Cloning of AtTRAPPC2L into the

	GGTC**GTT**CAAACCGTACGATCCAAC	pDONR201 vector
BP-C2w-F	GGGGACAAGTTTGTACAAAAAAGCA GGCTTC**ATG**GCTAACACTGCCTGC	Cloning of AtTRAPPC2 into the pDONR201 vector
BP-C2w-R	GGGGACCACTTTGTACAAGAAAGCTG GGTCCAGGTACTTTCTTGCAAG	Cloning of AtTRAPPC2 into the pDONR201 vector
C11pop-F	CACC**ATG**GAGGAATACCC	Cloning of AtTRAPPC11 into the pENTR/d-TOPO vector
TR-C11-R	CTTGCTGGTAGAGATGGC	Cloning of AtTRAPPC11 into the pENTR/d-TOPO vector
C12pop-F	CACC**ATG**GTATCTATTGGAAAGAC	Cloning of AtTRAPPC12 into the pENTR/d-TOPO vector
TR-C12-R	GACTCTGGTACAAGATGAATCAAAG	Cloning of AtTRAPPC12 into the pENTR/d-TOPO vector
C13-BP-F	GGGGACAAGTTTGTACAAAAAAGCA GGCTTC**ATG**AGCGCGACGCAGACG	Cloning of AtTRAPPC13 into the pDONR201 vector
C13-BP-R	GGGGACCACTTTGTACAAGAAAGCTG GGTC**GTC**TGTCTCTACAAATATCTC	Cloning of AtTRAPPC13 into the pDONR201 vector
C8-F3	CCTCCCAGCCAAACTGATGT	Colony PCR of yeast for Y2H assay and semi-quantitative PCR
C8-R4	GGACTTACCTGAACCGAGGG	Colony PCR of yeast for Y2H assay and semi-quantitative PCR
C11-F3	ACAAGCTCAGCAACTGGTCA	Colony PCR of yeast for Y2H assay
C11-R4	ACAACAACAGTTCCAGGGCA	Colony PCR of yeast for Y2H assay
C2L-F3	GAGGCTTTTCTTGGCTTGCTC	Colony PCR of yeast for Y2H assay
C2L-R4	CTTCCTGAAGAAACTTCTCACATCG	Colony PCR of yeast for Y2H assay
C2w-F1	ACCTAGCATGGACTACAAGTGC	Colony PCR of yeast for Y2H assay
C2w-R2	CATCAGTCGGGTATGGCCTG	Colony PCR of yeast for Y2H assay
C12-F3	GGGACGCATTACAGGTACGA	Colony PCR of yeast for Y2H assay
C12-R4	ACGATCTAACCCTTCCTGACG	Colony PCR of yeast for Y2H assay
C13-F3	TCCAATTGAATCTTATTGCCTCCA	Colony PCR of yeast for Y2H assay
C13-R4	ACTCGGGGAGGCTGTCTTAT	Colony PCR of yeast for Y2H assay
C8-F1	CAACCAAACGTAGAGGTTGCT	RT-qPCR analysis and semi-quantitative PCR

C8-R2	TACCGAAACCATGAGGGTGC	RT-qPCR analysis and semi-quantitative PCR
C8-F13	TGGTTCCATCGTAGGTGTGC	RT-qPCR analysis
C8-R14	CAAGCCTGGGTAGACTCTTGA	RT-qPCR analysis
CNX1-F	ATGAGACAACGGCAACTATTTTCC (Cho and Kanehara, 2017)	RT-qPCR analysis
CNX1-R	CCATAATCCTCATGTCCTTCACT (Cho and Kanehara, 2017)	RT-qPCR analysis
BiP1/2-F	GGTATCGAGACTGTAGGAGG (Cho and Kanehara, 2017)	RT-qPCR analysis
BiP1/2-R	GGTACGTTGTGAAAACCTGA (Cho and Kanehara, 2017)	RT-qPCR analysis
BiP3-F	CGAAACGTCTGATTGGAAGAA (Cho and Kanehara, 2017)	RT-qPCR analysis
BiP3-R	GGCTTCCCATCTTTGTTCAC (Cho and Kanehara, 2017)	RT-qPCR analysis
ERDJ3A-F	TCAAGTGGTGGTGGTTTCAACT (Pastor-Cantizano et al., 2018)	RT-qPCR analysis
ERDJ3A-R	CCCACCGCCCATATTTTG (Pastor-Cantizano et al., 2018)	RT-qPCR analysis
PDI6-F	CGAAGTGGCTTTGTCATTCCA (Pastor-Cantizano et al., 2018)	RT-qPCR analysis
PDI6-R	GCGGTTGCGTCCAATTTT (Pastor-Cantizano et al., 2018)	RT-qPCR analysis
bZIP60s-F	GGAGACGATGATGCTGTGGCT (Pastor-Cantizano et al., 2018)	RT-qPCR analysis
bZIP60s-R	CAGGGAACCCAACAGCAGACT (Pastor-Cantizano et al., 2018)	RT-qPCR analysis
NBR1_5	TGGTCGATACATTTCTTATTGGAGG (Jung et al., 2020)	RT-qPCR analysis
NBR1_6	GGGAGGCATTAAGGTTCAGTC (Jung et al., 2020)	RT-qPCR analysis

HMGR1-F	TCTATCGAGGTGGGGACAGT	RT-qPCR analysis
HMGR1-R	GATCGTCGCTAGCCTCCTTG	RT-qPCR analysis
HMGR2-F2	CCCAGCTCAAAACGTGGAGA	RT-qPCR analysis
HMGR2-R2	TCCCACCTCCAACAGTACCA	RT-qPCR analysis
MVK-F	ATGTGGCTCTCCTCTGGGAT	RT-qPCR analysis
MVK-R	CCAGACCCGTATGGAAGCTC	RT-qPCR analysis
DXS-F	GCGTGCTTATGACCAGGTTG	RT-qPCR analysis
DXS-R	ATGTGTCGGACCATCAGCTC	RT-qPCR analysis
DXR-F	TCCAGGAGAGCAAGGAGTGA	RT-qPCR analysis
DXR-R	TGCAGCAACCGTAGGCTTTA	RT-qPCR analysis
MCT-F	CGGTTGGAGCAGCTGTACT	RT-qPCR analysis
MCT-R	TCACCTGTGGTGTCTGCATT	RT-qPCR analysis
FPPS1-F	TAAACCCGACCCATCGAACG	RT-qPCR analysis
FPPS1-R	TTGGTGTCCCTCAATCGCTC	RT-qPCR analysis
FPPS2-F	TTGCTCATGGCGGGAGAAAA	RT-qPCR analysis
FPPS2-R	TGCCAAGTGTCTCAGGATCA	RT-qPCR analysis
AtCPT3_F	GCGCTTATGTCGATGCTG	RT-qPCR analysis
AtCPT3_R	CAGACTCAACCTCCTCAGG	RT-qPCR analysis
qPPR2F_2	GTCACGCCATTCTTGGATCG	RT-qPCR analysis for PPRD2
qPPR2R_2	TCGCAATCAGTAAACCCGCA	RT-qPCR analysis for PPRD2
PP2A-F	TAACGTGGCCAAAATGATGC	RT-qPCR analysis
PP2A-R	GTTCTCCACAACCGCTTGGT	RT-qPCR analysis

Cho, Y., & Kanehara, K. (2017). Endoplasmic reticulum stress response in Arabidopsis roots. *Frontiers in Plant Science*, *8*, 225027. https://doi.org/10.3389/FPLS.2017.00144/BIBTEX

Jung, H., Lee, H. N., Marshall, R. S., Lomax, A. W., Yoon, M. J., Kim, J., Kim, J. H., Vierstra, R. D., & Chung, T. (2020). Arabidopsis cargo receptor NBR1 mediates selective autophagy of defective proteins. *Journal of Experimental Botany*, *71*(1), 73–89. https://doi.org/10.1093/JXB/ERZ404

Pastor-Cantizano, N., Bernat-Silvestre, C., Marcote, M. J., & Aniento, F. (2018). Loss of Arabidopsis p24 function affects ERD2 trafficking and Golgi structure, and activates the unfolded protein response. *Journal of Cell Science*, *131*(2). https://doi.org/10.1242/JCS.203802/258327/AM/LOSS-OF-ARABIDOPSIS-P24-FUNCTION-AFFECTS-ERD2
