# Supplementary material for: The TRAPPC8/TRS85 subunit of the Arabidopsis TRAPPIII tethering complex regulates endoplasmic reticulum function and autophagy
Source: Plant Physiol. 2025 Mar 14;197(3):kiaf042. doi: 10.1093/plphys/kiaf042 (PMC11907232; doi:10.1093/plphys/kiaf042)
Supplement: kiaf042_Supplementary_Data [file pp.02624.2024-s05.pdf › pp.02624.2024-s05.pdf]

Plant Physiology®

The Arabidopsis thaliana TRAPPIII subunit AtTRAPPC8/AtTRS85 is involved in ER functions and autophagy

Marta Hoffman-Sommer, Natalia Pilka, Anna Anielska-Mazur, Julita Nowakowska, Małgorzata Kozieradzka-Kiszkurno, Cezary Pączkowski, Małgorzata Jemioła-Rzemińska, Kamil Steczkiewicz, Yasin Dagdas, and Ewa Swiezewska

Review Timeline:

Submission Date:	18-Jan-2024
Editorial Decision:	20-Mar-2024
Resubmission Received:	10-Oct-2024
Editorial Decision:	18-Oct-2024
Revision Received:	18-Nov-2024
Editorial Decision:	02-Dec-2024
Revision Received:	18-Dec-2024
Accepted:	20-Dec-2024

17-Mar-2024

Dr. Marta Hoffman-Sommer
Instytut Biochemii i Biofizyki Polskiej Akademii Nauk
Warsaw
Poland

RE: The Arabidopsis thaliana TRAPPIII subunit AtTRAPPC8/AtTRS85 is involved in ER functioning and autophagy

Dear Dr. Marta Hoffman-Sommer:

Thank you for giving us the opportunity to review your manuscript for publication in the Plant Physiology. The reviewers have found the manuscript of strong interest, but have raised a number of concerns that will require your attention. As these concerns are of some gravity and will entail substantial changes to the manuscript, you are likely to need additional time to address them. I must therefore reject the manuscript in its present form. Nonetheless, if you are able to address the points raised in review, I would welcome your resubmission of a manuscript that incorporates your responses.

The reviewers provided many specific and constructive comments that can be used to revise the paper. Of major importance is linking the molecular/cellular phenotypes as directly as possible with the organ-scale phenotyping to provide a solid foundation for future work on this protein complex function and plant development.

Please note that any resubmission will be treated as a new entry to the system and is likely to go out for review.

If you do have any queries or concerns, you are welcome to contact me in the first instance.

Thank you again for allowing Plant Physiology to review your work. We look forward to hearing from you soon.

Sincerely,

Editor, Plant Physiology

-- Reviewer comments:
Reviewer #1 (Comments for the Author):

MSID: PP2024-RA-00077

In this study, Hoffmann-Sommer et al. further our understanding of the TRAPPIII complex in Arabidopsis. Via yeast two-hybrid analysis, they confirm that the Arabidopsis TRAPPIII complex is similar - in terms of subunit composition and organization - to the metazoan complex. A careful phenotypic analysis of the TRAPPIII-specific subunit TRAPPC8 exhibits some interesting defects. These are interpreted as evidence for roles in autophagy, the ER stress response, secretion, and sorting. In addition, increased accumulation of dolichol is observed in the mutants.

The TRAPPIII complex is very important but poorly characterized in plants. Hence, this is a timely and important contribution.

The phenotypic analysis is carefully executed, the experiments overall are well performed. Very interesting are the experiments on autophagy and on the unfolded protein response (UPR).

The presentation is clear.

Major concerns:
1. The authors write about Rab "regulation" but fail to distinguish between upstream Rab activators (cognate GEFs) versus downstream effectors. This would help in

(i) the introduction lines 74-80: the statement that TRAPPII activates different Rab clades is incorrect. Indirect in vivo evidence suggests that TRAPPII activates Rab-A GTPases (evidence is that cognate GEFs preferentially bind GDP-bound Rab GTPases; and that constitutively activated RabA GTPases can suppress trappii phenotypes) but there is - to my knowledge - no evidence that AtTRAPPII can activate Rab-D, B, or E GTPases. Binding to these clades is shown in Kalde et al., 2019, but binding, even to a GEF, does not necessarily mean activation. See also Qi & Zheng 2011.
(ii) Fig. 11. Here the figure is misleading in that it also does not distinguish between the directionality of TRAPP-Rab interactions. See also comment 3.

2. The yeast-two hybrid is a very timely experiment, and the results are conclusive. However, it would be important to perform the three following analyses on the Arabidopsis TRAPPIII subunits:
a) AlphaFold structural predictions
b) a documentation of plant-specific versus conserved domains wrt metazoan and yeast TRAPPIII
c) cross-kingdom structural alignments against metazoan TRAPPIII (see for example 10.1101/2023.04.24.537966 figure S4)
3. Fig. 11 is misleading in that it suggests that TRAPPC8 localizes to its cellular sites of action. The figure is difficult to reconcile with TRAPPC8 and TRAPPC11 localization to the TGN/EE, as shown in previous studies. It may be safer to highlight the different trafficking STEPS (as opposed to compartments) that are impacted in trappc8 mutants, and then candidly discuss how this can be interpreted in light of the TGN/EE localisation of the subunits. Of course, localisation is not function.
4. Rather than emphasize the similarities between Arabidopsis and metazoan TRAPPPIII, the authors could discuss the unique demands of endomembrane traffic in plants, and how TRAPPIII might support these.

Overall: this is a timely and important study because the TRAPPIII complex is very important but poorly understood in plants. What would be important for or needed by the reader is a more extensive tabulation (including reference to the different names of the subunits) and visualization of the subunit composition of the complex (including reference to the literature). The tabulation could extend to the function and localization of the subunits in different organisms.
If presented in such a way as to help the reader cope with the apparent gaps in the literature, this study would comprise a much needed story of interest to the broad readership of Plant Phys.

Reviewer #2 (Comments for the Author):

This manuscript by Hoffman-Sommer et at. reports many interesting phenotypes of an Arabidopsis mutant defective in a component of TRAPPIII called C8 (also called Trs85 in Allen et al. 2024, Plant J). They show that the mutant is defective in basal but not stressed-induced autophagy. The mutant is hyposensitive to Tm, a drug induces ER stress. There is an accumulation of Dolichol, an ER lipid carrying the precursor of N-glycan. The major weakness of the manuscript is the linkage between different phenotypes described or molecular/cellular basis for all described. The authors spent a section to discuss possible roles of C8 with Rabs, but no data presented (except cell developmental phenotypes) in the manuscript for how C8 may interplay with Rabs.

It is highly possible that C8 plays multiple roles in protein transport with the endomembrane system and autophagy. I suggest authors to focus on possible roles of C8 in autophagy and/or ER accumulations of ER proteins and/or lipids (not reported previously at all) to make a step forward on how C8 may participate autophagy and ER functioning, which may count at least in part for what Song et al. (2020) reported.

Some suggestions:
1. A molecular or genetic complementation is needed to confirm phenotypes described is caused by mutations in C8.
2. Was the localization of C8 reported in Drakakaki et al., 2012?
3. How NBR1 and Atg8 may behave in nutrient starvation conditions? Can authors investigate how Atg9 may cycle in basal and stress-induced conditions in the mutant?
4. Do ER proteins accumulate in the mutant in additional to Dolichol? How does the ER look like in the mutant?
5. Potential interactions within the TRAPPIII complex. Y2H was used. However, due to the inherent limitations of this heterologous expression system, notably false positives and negatives, it is advisable that the conclusions drawn to be confirmed by alternative methods.

Minor issues:
1. To better elucidate the structure of the potential plant TRAPPIII complex, it is recommended that authors create a diagrammatic representation to visually convey this information.
2. Line 90: should be "analyses".
3. The elevated fold of NBR1 protein level should be quantified to compare with the elevated mRNA in the trappc8 mutant.
4. Line 380: "not shown" should not be used in a publication nowadays.
5. Fig 2 and elsewhere: Only t-test used for statistical analyses. Multiple comparisons tests should be used for three or more comparisons.

We thank the reviewers for comments and suggestions that were truly constructive and helped us improve the manuscript. Answers to specific comments are presented below.

Reviewer #1

Major concerns:

1. The authors write about Rab "regulation" but fail to distinguish between upstream Rab activators (cognate GEFs) versus downstream effectors. This would help in
(i) the introduction lines 74-80: the statement that TRAPPII activates different Rab clades is incorrect. Indirect in vivo evidence suggests that TRAPPII activates Rab-A GTPases (evidence is that cognate GEFs preferentially bind GDP-bound Rab GTPases; and that constitutively activated RabA GTPases can suppress trappii phenotypes) but there is - to my knowledge - no evidence that AtTRAPPII can activate Rab-D, B, or E GTPases. Binding to these clades is shown in Kalde et al., 2019, but binding, even to a GEF, does not necessarily mean activation. See also Qi & Zheng 2011.
(ii) Fig. 11. Here the figure is misleading in that it also does not distinguish between the directionality of TRAPP-Rab interactions. See also comment 3.

We acknowledge that the interactions of TRAPP complexes with Rab proteins were discussed in a misleading way. We have re-written parts of the Introduction, leaving out some of the information on the relation with Rab proteins because we decided they were not relevant for the rest of the paper. We also modified the scheme in Figure 11 and we hope that it no longer suggests direct localization of TRAPPC8 to its sites of function.

2. The yeast-two hybrid is a very timely experiment, and the results are conclusive. However, it would be important to perform the three following analyses on the Arabidopsis TRAPPIII subunits:
a) AlphaFold structural predictions
b) a documentation of plant-specific versus conserved domains wrt metazoan and yeast TRAPPIII
c) cross-kingdom structural alignments against metazoan TRAPPIII (see for example 10.1101/2023.04.24.537966 figure S4)

Thank you for this comment. We performed the suggested experiments and we present the results in Figures 1 and S2. Relevant observations are summarized in the Results section and also commented in the Discussion.

3. Fig. 11 is misleading in that it suggests that TRAPPC8 localizes to its cellular sites of action. The figure is difficult to reconcile with TRAPPC8 and TRAPPC11 localization to the TGN/EE, as shown in previous studies. It may be safer to highlight the different trafficking STEPS (as opposed to compartments) that are impacted in trappc8 mutants, and then candidly discuss how this can be interpreted in light of the TGN/EE localisation of the subunits. Of course, localisation is not function.

The reviewer is absolutely right, thinking through their comments has allowed us to modify Figure 11 accordingly. We hope this version appears more clear.

4. Rather than emphasize the similarities between Arabidopsis and metazoan TRAPPPIII, the authors could discuss the unique demands of endomembrane traffic in plants, and how TRAPPIII might support these.

Parts of the Discussion have been re-written in an attempt to emphasize this.

Overall: this is a timely and important study because the TRAPPIII complex is very important but poorly understood in plants. What would be important for or needed by the reader is a more extensive tabulation (including reference to the different names of the subunits) and visualization of the subunit composition of the complex (including reference to the literature). The tabulation could extend to the function and localization of the subunits in different organisms.
We are very pleased that the Reviewer is positive about the direction of our research. Following the suggestion, we included a scheme of TRAPPIII and a table with subunit names in modified Figure 1.

Reviewer #2
Suggestions:
1. A molecular or genetic complementation is needed to confirm phenotypes described is caused by mutations in C8.
Our attempts to generate complemented lines were unsuccessful so far, so we decided to cross *trappc8-1* and *trappc8-2* plants and analyze the phenotype of the homozygotic double mutants thus obtained. The phenotypic features of these double mutant plants, now shown in Figure S4, document that the mutations *trappc8-1* and *trappc8-2* are allelic.

2. Was the localization of C8 reported in Drakakaki et al., 2012?
Thank you for this important question – AtTRAPPC8 was found on TGN SYP61-containing vesicles by Drakakaki et al. This is now mentioned in line 105 of the Introduction.

3. How NBR1 and Atg8 may behave in nutrient starvation conditions? Can authors investigate how Atg9 may cycle in basal and stress-induced conditions in the mutant?
Following the suggestions of Reviewer #2, we decided to investigate more deeply one of the aspects of TRAPPC8 biology, namely the ER phenotype and its morphological features as well as the level of accumulation of selected lipids (phytosterols and fatty acids). New data summarizing these experiments are presented in Figures 9 and 10.

4. Do ER proteins accumulate in the mutant in additional to Dolichol? How does the ER look like in the mutant?
Thank you for raising this question which prompted us to observe ER morphology by TEM microscopy (Figure 9) - we found that the morphology is unchanged. In parallel, we performed a test for plant sensitivity to DTT (general ER stress agent) and found that DTT sensitivity was not alleviated by the *trappc8* mutations to the extent that tunicamycin sensitivity is (Figure 9). We also assayed further lipids present in the ER (sterols, fatty acids) and found that their levels are not changed in the mutants (Figure 10). These observations reinforce our conclusions that the changes in dolichol levels are specific for this lipid and influence the potential of the cells to cope with glycosylation-related stress.

5. Potential interactions within the TRAPPIII complex. Y2H was used. However, due to the inherent

limitations of this heterologous expression system, notably false positives and negatives, it is advisable that the conclusions drawn to be confirmed by alternative methods.

The subunits assayed here by Y2H have been previously co-precipitated by others (Rosquete et al. 2019b, Kalde et al. 2019, Garcia et al. 2020), and here we made an attempt to verify the selected direct interactions that would shed light on the architecture of the complex. We now strengthen the conclusions by including structural modeling which corresponds well with the Y2H data. We hope the approach currently presented is more convincing.

Minor issues:

1. To better elucidate the structure of the potential plant TRAPPIII complex, it is recommended that authors create a diagrammatic representation to visually convey this information.

A scheme has been included in Figure 1.

2. Line 90: should be "analyses".

We are grateful for spotting this. The sentence has been now re-written in the new version of the Introduction (lines 81-82).

3. The elevated fold of NBR1 protein level should be quantified to compare with the elevated mRNA in the trappc8 mutant.

It is now impossible to quantify the bands on the Western blot in a convincing manner because some of the bands are oversaturated. For this reason we decided the experiment was not very conclusive and moved it to the Supplementary materials (discussed in lines 719-723).

4. Line 380: "not shown" should not be used in a publication nowadays.

The mentioned data is now shown in Figure S1.

5. Fig 2 and elsewhere: Only t-test used for statistical analyses. Multiple comparisons tests should be used for three or more comparisons.

We would like to thank the Reviewer for raising this point, however in fact we do not perform multiple comparisons tests in this study, because we are only comparing *pre-selected* data columns, not searching for significant differences between *all* columns. For this reason the unpaired t-test with Welch's correction was employed – we believe this approach satisfies the requirements of the experimental approach we used.

18-Oct-2024

Dr. Marta Hoffman-Sommer
Instytut Biochemii i Biofizyki Polskiej Akademii Nauk
Laboratory of Lipid Biochemistry
Warsaw
Poland

RE: The Arabidopsis thaliana TRAPPIII subunit AtTRAPPC8/AtTRS85 is involved in ER functioning and autophagy

Dear Dr. Marta Hoffman-Sommer:

We are pleased to accept your manuscript for publication in the Plant Physiology. This acceptance is contingent on revision based on the comments of our reviewers. In particular, please address the editorial suggestions of both reviewers in the final resubmitted version of the paper. I would also suggest a minor edit to the title to make it more active in voice.

Change to: "The Arabidopsis thaliana TRAPPIII subunit AtTRAPPC8/AtTRS85 is involved in ER functions and autophagy"

Please highlight all changes and include a detailed annotation to changes to the text, with line numbers, and noting your responses to the comments.

To submit your revised manuscript, click:

Link Not Available

If you cannot return the revised manuscript within 8 weeks of receipt, please let us know. Otherwise, we will assume that you have elected not to revise the manuscript and are withdrawing it.

Thank you for allowing us to review your work. We look forward to hearing from you soon.

Sincerely,

Editor, Plant Physiology

-- Reviewer comments:
Reviewer #1 (Comments for the Author):

This is a revised version of an earlier submission. The authors have substantially improved their manuscript. Figures 1A and 1D (new) are very helpful for the reader, as is Figure 11 (revised).
I have only two comments about the introduction and discussion: The authors write:

1. Introduction lines 101-103: "Plant TRAPPs are not as well described as the yeast and metazoan complexes. Their subunit composition has been analyzed (Kalde et al., 2019; Garcia et al., 2020), but no structural data has been collected yet and their substrate specificity also remains uncertain."
This is not entirely true: for TRAPPII, higher order structural alignments (based on alpha-fold structural predictions) have been carried out (Wiese et al., 2024), as well as extensive pairwise yeast two-hybrid experiments (Kalde et al., 2019). Furthermore, there is indirect in vivo evidence for Rab-A GEF activity (see Kalde et al., 2019 for data and discussion/review of previous literature). Seeing as the manuscript by Hoffman-Sommer et al., does not provide direct structural data, GEF assays, differential binding to GDP or GTP-bound Rab GTPases or mutant rescue via GTP-bound RAB GTPases, it seems misleading to word things this way. Hoffman-Sommer et al. provide alpha-fold structural predictions of TRAPPIII, as well as careful mutant analyses.
I suggest the introduction state:
Plant TRAPPs are not as well described as the yeast and metazoan complexes. Their subunit composition has been analyzed (Kalde et al., 2019; Garcia et al., 2020), but open questions remain regarding their putative GEF functions. Furthermore, no structural data has been collected for Arabidopsis TRAPPIII to date.

2. Discussion lines 1038-1040
"The TRAPPIII and -II complexes share a seven-subunit core structure and have at least partially overlapping localization. It has long been known that the TRAPP core is sufficient for GEF activity (Sacher et al., 2001) with the TRAPPIII and -II specific subunits serving regulatory functions."
Sacher et al. 2001 is dated, for TRAPPII the authors should refer to recent publications showcasing the Rab-GTPase binding

site and GEF enzyme pocket, where TRAPPII-specific subunits appear to play a more prominent possible role than initially suspected.

Reviewer #2 (Comments for the Author):

The authors addressed my questions/concerns in this revised manuscript. Two minor issues:

1. Lines 708-709: 'Judged by the extent of leaf chlorosis occurring, the trappc8 mutants were slightly sensitive to nitrogen starvation, though not to carbon starvation (Fig. 8A)'. A quantification for the sensitivity of the mutant to nitrogen and carbon starvation should be provided to justify the description.

2. Fig. 9B. Fragmented vacuoles and the ER should be pointed out in Fig. 9B.

Changes to manuscript and replies to Reviewers – revision of manuscript PP2024-RA-02624D by Marta Hoffman-Sommer et al.

We thank the Reviewers for the comments, all answers are presented below.

Reviewer #1

Q1: Introduction lines 101-103: "Plant TRAPPs are not as well described as the yeast and metazoan complexes. Their subunit composition has been analyzed (Kalde et al., 2019; Garcia et al., 2020), but no structural data has been collected yet and their substrate specificity also remains uncertain."
This is not entirely true: for TRAPPII, higher order structural alignments (based on alpha-fold structural predictions) have been carried out (Wiese et al., 2024), as well as extensive pairwise yeast two-hybrid experiments (Kalde et al., 2019). Furthermore, there is indirect in vivo evidence for Rab-A GEF activity (see Kalde et al., 2019 for data and discussion/review of previous literature). Seeing as the manuscript by Hoffman-Sommer et al., does not provide direct structural data, GEF assays, differential binding to GDP or GTP-bound Rab GTPases or mutant rescue via GTP-bound RAB GTPases, it seems misleading to word things this way. Hoffman-Sommer et al. provide alpha-fold structural predictions of TRAPPIII, as well as careful mutant analyses.
I suggest the introduction state:
Plant TRAPPs are not as well described as the yeast and metazoan complexes. Their subunit composition has been analyzed (Kalde et al., 2019; Garcia et al., 2020), but open questions remain regarding their putative GEF functions. Furthermore, no structural data has been collected for Arabidopsis TRAPPIII to date.

A1: We thank for precise wording of this issue. We agree that the formulation suggested by the Reviewer describes the current state of knowledge much better than the original sentence. We have modified the lines accordingly (lines 101-104).

Q2: Discussion lines 1038-1040
"The TRAPPIII and -II complexes share a seven-subunit core structure and have at least partially overlapping localization. It has long been known that the TRAPP core is sufficient for GEF activity (Sacher et al., 2001) with the TRAPPIII and -II specific subunits serving regulatory functions."
Sacher et al. 2001 is dated, for TRAPPII the authors should refer to recent publications showcasing the Rab-GTPase binding site and GEF enzyme pocket, where TRAPPII-specific subunits appear to play a more prominent possible role than initially suspected.

A2: The paper by Sacher et al. (2001) was mentioned in an attempt to make it clear to the reader that data on in vitro GEF activity has been available for a long time. However, the Reviewer is absolutely right that the manuscript should refer to current literature data that describes the most recent advances in structural investigations of TRAPP complexes. We reformulated the lines (now 1041-1045) to incorporate a more updated view of the role of the complex-specific subunits, trying at the same time not to make the text any longer:

“The TRAPPIII and -II complexes share a seven-subunit core structure and have at least partially overlapping localization. The complex core is indispensable for GEF activity, but also the TRAPPIII and -II

specific subunits are involved in efficient Rab binding and nucleotide exchange (reviewed in Sun & Sui, 2023), and they determine the transition between GEF activity towards Rab proteins of different families, serving also a regulatory role in the complexes.”

Reviewer #2

Q1: Lines 708-709: 'Judged by the extent of leaf chlorosis occurring, the trappc8 mutants were slightly sensitive to nitrogen starvation, though not to carbon starvation (Fig. 8A)'. A quantification for the sensitivity of the mutant to nitrogen and carbon starvation should be provided to justify the description.

A1: Following the Reviewer’s suggestion, we performed semi-quantitative assessment of leaf bleaching using ImageJ. The data was normalized to the average intensity of leaves from the WT control and the resulting graphs are shown below:

Carbon starvation:

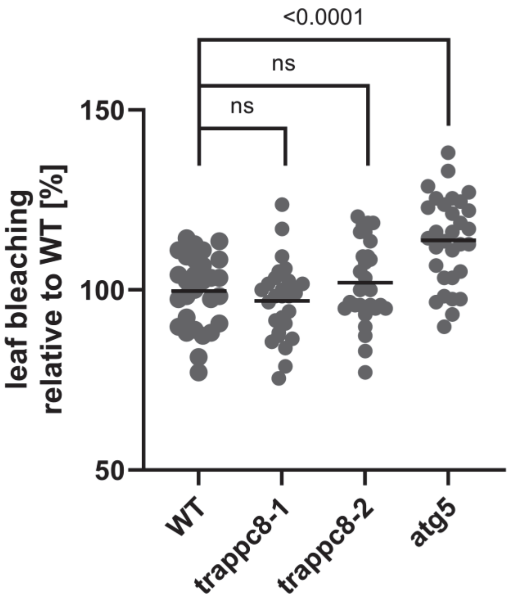

Nitrogen starvation:

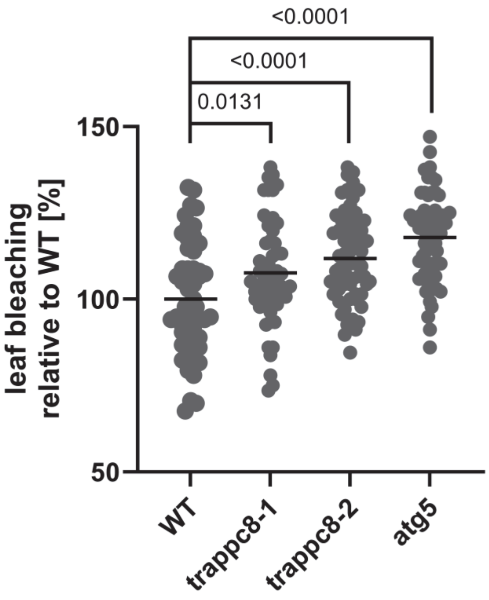

In our opinion the results of this semi-quantitative analysis do not provide any additional insight into the observed phenomenon. Still, they are in line with the qualitative judgment summarized in the text: “Judged by the extent of leaf chlorosis occurring, the *trappc8* mutants were slightly sensitive to nitrogen starvation, though not to carbon starvation (Fig. 8A)” (line 710).

We do not consider this analysis of sufficient value to be presented in the article. We kindly ask the Reviewer if she / he is satisfied with this reasoning, otherwise we could include this semi-quantitative data analysis as part of the Supplementary Material.

Q2: Fig. 9B. Fragmented vacuoles and the ER should be pointed out in Fig. 9B.

A2: Arrows have been included on the appropriate panels and described in the caption of Figure 9 (lines 839-840).

Other minor changes introduced:

Line 2 and 22 (title and running title), and lines 956 and 1055: We modified the title as suggested by the Editor.

Line 13-14: We made a correction to the affiliation of one of the Authors.

Lines 303-305: We added a sentence to the TEM method section (previously omitted by mistake).

Lines 1091-1096: We included a Data Availability statement.

02-Dec-2024

Dr. Marta Hoffman-Sommer
Instytut Biochemii i Biofizyki Polskiej Akademii Nauk
Laboratory of Lipid Biochemistry
Warsaw
Poland

RE: The Arabidopsis thaliana TRAPPIII subunit AtTRAPPC8/AtTRS85 is involved in ER functions and autophagy

Dear Dr. Marta Hoffman-Sommer:

We are pleased to accept your manuscript for publication in the Plant Physiology. Thank you for your thoughtful and thorough responses to the reviewer comments. The paper is acceptable in its current form, just add the quantitative data on the nitrogen sensitivity of the mutants as a supplemental figure to back up the statements in the paper.

Please make these small changes and highlight a detailed annotation to changes to the text, with line numbers, and noting your responses to the comments. I will then quickly approve the final version.

To submit your revised manuscript, click:

Link Not Available

If you cannot return the revised manuscript within 8 weeks of receipt, please let us know. Otherwise, we will assume that you have elected not to revise the manuscript and are withdrawing it.

Thank you for allowing us to review your work. We look forward to hearing from you soon.

Sincerely,

Editor, Plant Physiology

-- Reviewer comments:

Thank you for reviewing our manuscript, entitled: „The *Arabidopsis thaliana* TRAPPIII subunit AtTRAPPC8/AtTRS85 is involved in ER functions and autophagy" (manuscript ID: **PP2024-RA-02624DR2**). The following changes have been made in the manuscript:

Line 710: a reference to Figure S6A has been added
Line 717: Figure S6 has been renamed to S6B
Lines 1094-1096: full doi's for datasets have been included
Supplementary Figures file, page 8: Figure S6 has been modified. It now includes two panels, with panel S6A presenting the quantitative analysis of the starvation plate assay presented in Figure 8A. Panel S6B represents the previous Figure S6 (not changed). The figure caption was also modified accordingly.

20-Dec-2024

Dr. Marta Hoffman-Sommer
Instytut Biochemii i Biofizyki Polskiej Akademii Nauk
Laboratory of Lipid Biochemistry
Warsaw
Poland

MSID: PP2024-RA-02624DR2
MS TITLE: The Arabidopsis thaliana TRAPPIII subunit AtTRAPPC8/AtTRS85 is involved in ER functions and autophagy

Dear Dr. Marta Hoffman-Sommer:

I am pleased to inform you that your revised manuscript "The Arabidopsis thaliana TRAPPIII subunit AtTRAPPC8/AtTRS85 is involved in ER functions and autophagy" has been accepted for publication in Plant Physiology. Your manuscript will be checked for consistency with journal formatting and image standards by one of our Science Editors, who will contact you shortly. They may have a number of queries and suggestions for you. Please keep in mind that they work to increase the online visibility of your article and its attraction for readers from the widest of backgrounds. You will have the opportunity to review any revisions, and I hope you will work constructively with them to get the most out of your research. You will also have the opportunity to review all changes when you receive the author proofs for your article.

Once validated for publication, your article will be sent to a Science Editor for a final review of your manuscript and source files. After your final files are uploaded, your article will appear in the next available issue and the online Preview version of your article will be posted after your signed Copyright and Checklist forms and final source files are received.

Please be sure to include the following in your manuscript file:
Harvard style citations
List of all author contributions and funding information at the beginning of manuscript after the titles and author list.
List all supplemental materials associated with the article, with titles, at the end of the ms, before the references and acknowledgments section.
Complete Figure Legends for all figures appearing in the article listed before the Literature Cited.

LICENSE INFORMATION Shortly after your paper is sent to production, you will receive an email from our publisher's author support team, **SciPris**, with information on article processing charges, as well as the types of Open Access license you would be able to purchase. The accepted version of your manuscript will not appear on our Advance Articles page until the license is selected and signed, so please look out for an email from SciPris. For more information see the Article Publication Fees section of our author guidelines: https://academic.oup.com/plcell/pages/General-Instructions

Finally, we encourage your submission of artwork for the journal cover. If there is an image or illustration related to your paper that you would like to have considered, please email it in .pdf or .tif format to clowe@aspb.org. An image title, brief, 2-3 sentence description of the image, and the name of the person credited for the image are also encouraged. If your image is selected to appear on the online cover of the journal, you will be contacted with the scheduled issue date and further information.

Thank you and congratulations on your Plant Physiology paper!

Sincerely,
Editor, Plant Physiology

=====
FIRST AUTHOR PROFILES
=====

We recognize the hard work that goes into being First Author of a Plant Physiology paper by publishing a "First Author Feature" for each First Author alongside your paper.

If the first author(s) would like to submit information for a feature on Facebook and Twitter, fill out the form by clicking on the link below:

Plant Physiology First Author Feature Form: https://forms.office.com/r/ZBCGZfhm8F
If you cannot access the form through the link above, please contact Rachel at rbelsky@aspb.org.

To upload an image of yourself to accompany your profile, please put your photo in the correct journal folder at the link below. You must label your image: Author First Name Last Name Article Title.

First Author Profile Image Dropbox: https://www.dropbox.com/scl/fo/xz029p7qjr2jml2rp2kmq/h?dl=0&rlkey=6fkxcaqmxx35ap152b2osy1fz

=====
IMPORTANT REMINDER: PEER REVIEW REPORTS
=====

If you opted to publish a peer review report along with your article during the original submission process, it will be prepared by the editorial staff and publicly posted with your manuscript, inside the zip file that contains any other supplemental material. As a reminder, the peer review report is a public record of all comments from editors and reviewers, as well as your prior responses, as you received them in the decision letters for each draft of your manuscript. If you agreed to publish this report and have changed your mind, or are not sure if you selected this option, please contact the editorial office as soon as possible before signing the license agreement from our publisher.

-- Reviewer comments:
